# Supplementary material for: IL-7R signaling activates widespread VH and DH gene usage to drive antibody diversity in bone marrow B cells
Source: Cell Rep. 2021 Jul 13;36(2):109349. doi: 10.1016/j.celrep.2021.109349 (PMC8293627; doi:10.1016/j.celrep.2021.109349)
Supplement: Document S1. Figures S1–S4 and Table S3 [file mmc1.pdf]

Cell Reports, Volume *36*

Supplemental information

IL-7R signaling activates widespread V_H and D_H gene usage to drive antibody diversity in bone marrow B cells

Amanda Baizan-Edge, Bryony A. Stubbs, Michael J.T. Stubbington, Daniel J. Bolland, Kristina Tabbada, Simon Andrews, and Anne E. Corcoran

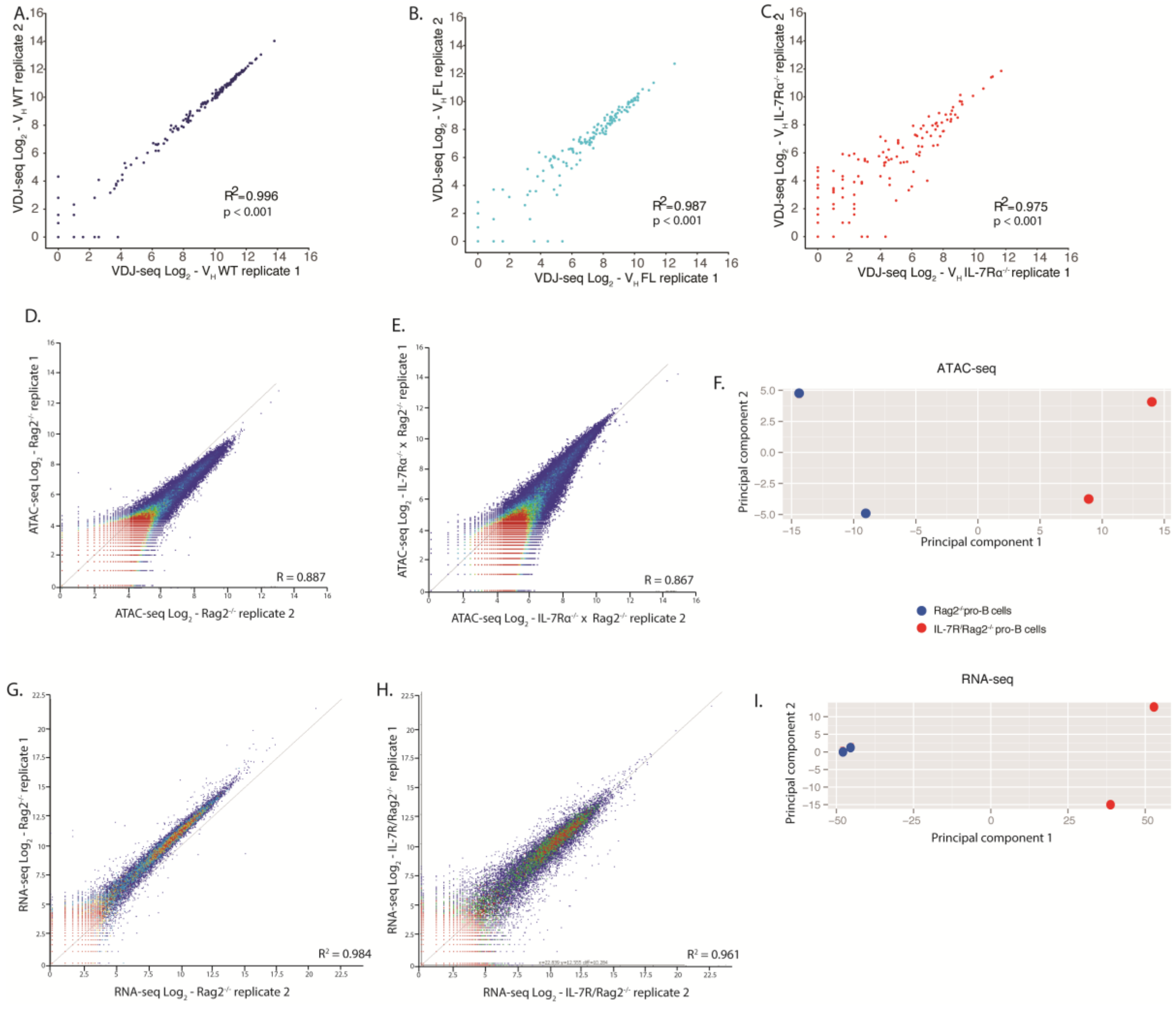

S1: Replicate VDJ-seq, ATAC-seq and RNA-seq libraries are highly correlated. Related to Figure 1 (panels A-C), 2 (panels A-C) and 3 (panels D-F and G-I).

A-C. Scatterplot of VDJ-seq read counts (log2 transformed) of V_H genes in two **A.** WT, **B.** FL and **C.** IL-7Rα$^{-/-}$ pro-B cell biological replicate datasets.

D-F. ATAC-seq read peaks were identified with MACS peak caller in Rag2$^{-/-}$ and IL-7Rα$^{-/-}$/Rag2$^{-/-}$ genotypes. Reads were quantified for peak probes for each biological replicate separately for **D.** Rag2$^{-/-}$ (Pearson's correlation R = 0.887) and **E.** IL-7Rα$^{-/-}$/Rag2$^{-/-}$ (R = 0.867) pro-B cell libraries. **F.** Principal component analysis shows clustering of each genotype. Principal components are calculated based on the regularized-logarithm transformation of read counts per gene and using the R package pcaMethods. Analysis done using DESeq2 package.

G-I. RNA-seq reads were quantified over all genes for each biological replicate for **G.** Rag2$^{-/-}$ and **H.** IL-7Rα$^{-/-}$/Rag2$^{-/-}$ pro-B cell libraries. **I.** Principal component analysis shows clustering of each genotype, calculated as for **F.** above.

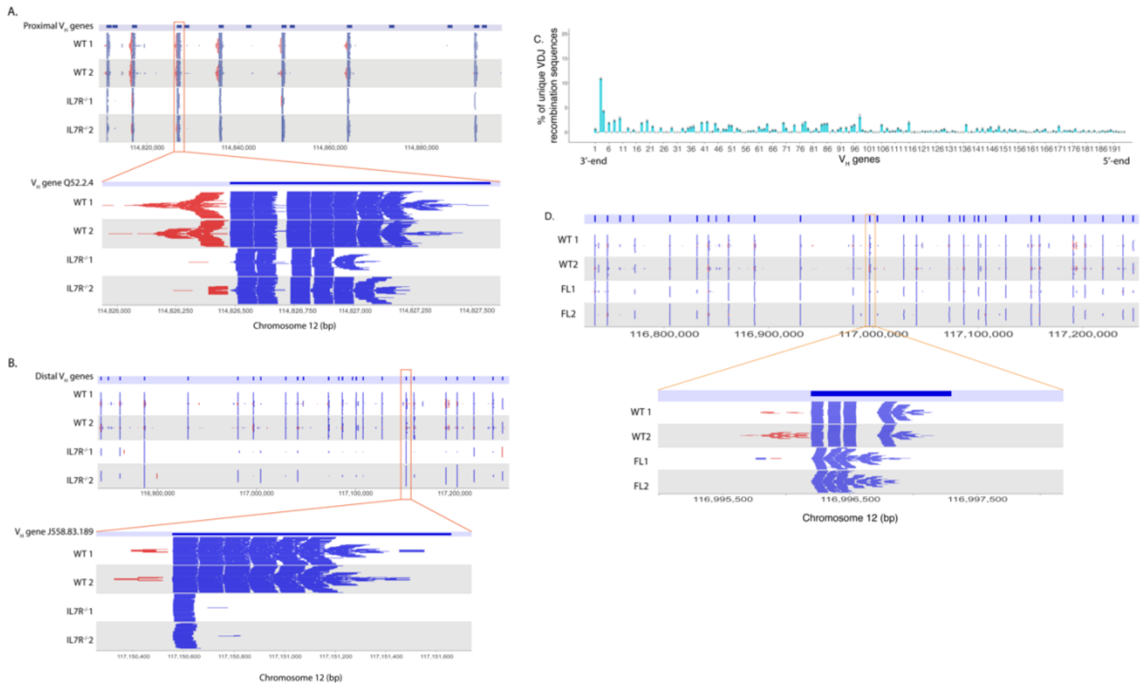

S2: VDJ-seq read depth view over the Igh V region. Related to Figure 1 and 2. Genome browser view of raw reads for each replicate WT and IL-7Rα$^{-/-}$ VDJ-seq library over the **A.** proximal V_H gene region (V_H genes in dark blue, starting with 7183.1pg.1 and 7183.8pg.14 as the most right) with a zoomed in view of Q52.2.4; and of the **B.** distal V_H gene region (the most left V_H genes represents J558.71pg.172 and the most right is J558.89pg.195) with a zoomed in view of J558.83.189. Due to the low number of cells in IL-7Rα$^{-/-}$ BM, VDJ-seq libraries were generated with approximately 6-fold less starting material than WT libraries, resulting in reduced numbers of raw reads (S Table 1). Nevertheless, this amount of starting material does not compromise detection of the wide dynamic range of frequency of VDJ and DJ recombined sequences (Chovanec et al., 2018). Blue reads = reverse, red reads = forward. Only correctly orientated (reverse) reads were quantified for VDJ-seq analysis.

C. Recombination frequencies of the 195 V_H genes measured by VDJ-seq, for FL pro-B cells from 15.5 day old wild-type mice (50 embryos per replicate). Two replicates are shown as open circles. Reverse-strand reads were quantified for each V_H gene and shown as a percentage of total number of reads quantified. For list of V_H genes see S Table 1. **D.** Genome browser view of raw reads for each replicate WT and FL VDJ-seq library over the distal V_H gene region (the most left V_H genes represents J558.66.165 and the most right is J558.89pg.195) with a zoomed in view of J558.75.177. Each WT and FL replicate is shown separately. For read depth mapped to each Igh region see S Table 1.

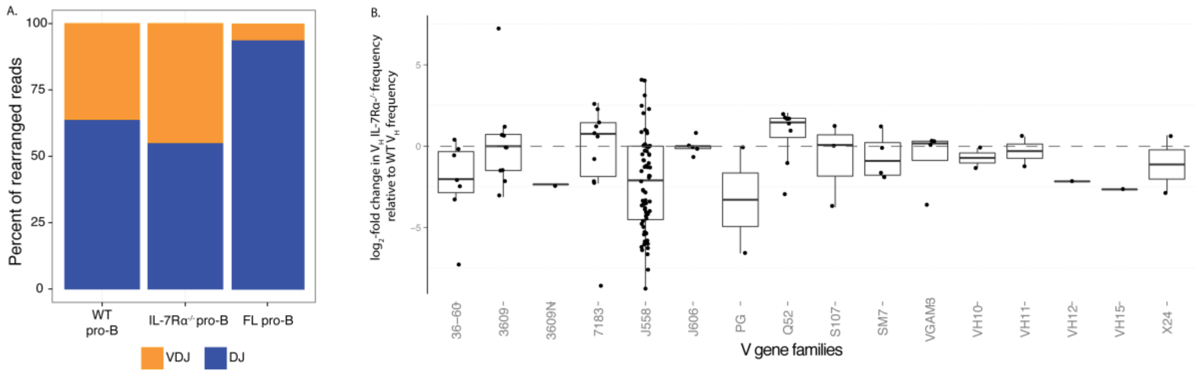

S3: DJ_H -VDJ_H ratio and individual V_H gene family usage. Related to Figure 1 and 2

A. Proportion of DJ_H and VDJ_H sequences in each cell type. The number of reads in the correct orientation was calculated over the D_H and V_H regions, and is shown as a percentage of total VDJ-seq reads in the correct orientation (mean of two replicates). **B.** VDJ-seq values of each V_H gene grouped in families. The average of two WT and two IL-7Rα$^{-/-}$ VDJ-seq replicates was calculated for each V_H gene. To display changes between WT and IL-7R-modified model frequencies, V_H frequencies for each model were divided with the WT mean value and log2-transformed for comparison between models. Log2 values for each gene were grouped by gene family.

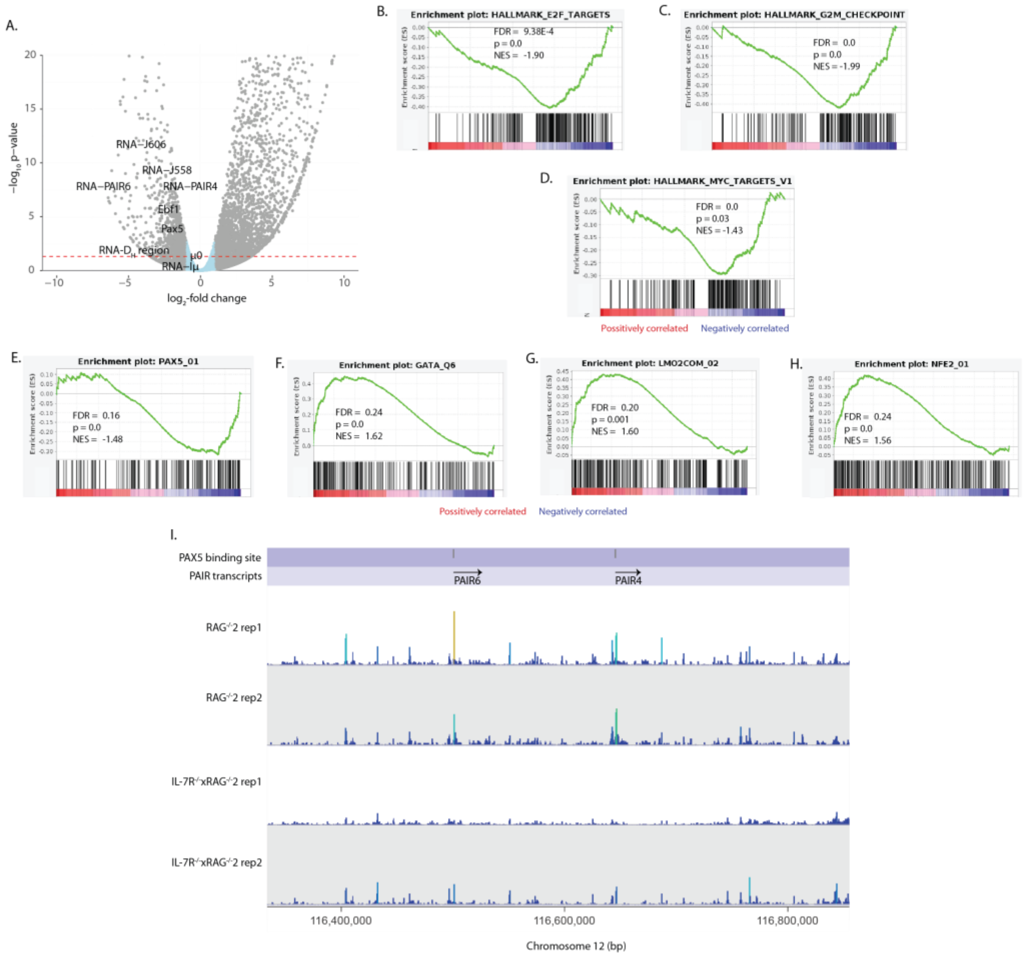

S4: Transcriptional changes in IL-7Rα/Rag2$^{-/-}$ pro-B cell libraries relative to Rag2$^{-/-}$. Related to Figure 4. **A.** Volcano plot showing genome-wide RNA-seq data. Grey dots are genes that are differentially expressed > 2-fold (in blue < 2-fold) in IL-7Rα$^{-/-}$/Rag2$^{-/-}$ compared with Rag2$^{-/-}$ libraries; dashed red line shows where p = 0.05. Transcripts of interest are tagged by name. Out of 4,793 differentially expressed genes in IL-7Rα/Rag2-/- pro-B compared with Rag2-/- pro-B cells, 3,780 genes were upregulated, while 1,013 genes were downregulated.

B-D. Gene set enrichment plot for **B.** E2F, **C.** G2M and **D.** MYC pathway target genes from the hallmark gene sets. Green line = enrichment score. FDR, false discovery rate; p, p-value; NES, normalized enrichment score.

E-H: Transcriptional profiles for lineage-specific TF in IL-7Rα$^{-/-}$/Rag2$^{-/-}$ pro-B cell. Gene set enrichment plot for **E.** PAX5, **F.** GATA, **G.** LMO2 and **H.** NFE2 pathway target genes from the transcription factor targets/regulatory target gene sets. Green line = enrichment score. FDR, false discovery rate; p, p-value; NES, normalized enrichment score.

I: PAIR4 and PAIR6 PAX5 binding sites have decreased accessibility in IL-7Rα$^{-/-}$Rag2$^{-/-}$ cells. ATAC-seq reads for Rag2$^{-/-}$ and IL-7Rα$^{-/-}$Rag2$^{-/-}$ were quantified per 500 bp bins along the Igh locus and normalised by reads per million in Seqmonk. Height and colour of each bar represents relative number of reads over each probe: high orange bars have more reads than short blue bars. Each track was generated from each ATAC-seq library replicate. Purple tracks represent the location of PAX5 binding sites (top) and the location of PAIR4 and PAI6 transcripts (black arrows, bottom).

Differentially expressed genes

Gene	$Rag2^{-/-}$ mean	IL-7Rα/$Rag2^{-/-}$ mean	log2 FoldChange	p-adj
Ebf1	198467.7	31632.21	-2.57	2.79E-07
Pax5	22013.66	5049.33	-2.05	5.39E-04
Flt3	371.14	2680.54	2.8	1.31E-14
Irf8	1727.72	7817.74	2.15	1.34E-09
Ikzf3	453.74	2896.93	2.58	6.89E-06
Lef1	50047.29	7152.81	-2.69	1.39E-05
Foxo1	19360.34	5176.08	-1.86	2.37E-05
Cd79a	36517.38	5427.5	-2.6	2.64E-04
Rag1	24672.56	4710.95	-2.26	2.37E-03
Smarca4	94899.66	25252.66	-1.84	3.19E-03
Ezh2	21004.9	8795.46	-1.23	3.77E-03
Myb	29838.38	16868.3	-0.8	2.41E-01
Irf4	5144.04	2515.98	-0.98	3.14E-01
Pdcd1	0	2.37	0.86	7.68E-01

Pax5 Igh

Gene	base Mean	log2Fold Change	stat	pvalue	padj	RAG2ko rep 1	RAG2ko rep 2	IL7RxRAG rep 1	IL7RxRAG rep 2	Start	End
PAX5_peak_4991	58.65	-1.607	-3.907	9.34E-05	0.003	104.98	71.196	21.862	36.603	116500485	116500825
PAX5_peak_4995	47.76	-1.757	-3.892	9.93E-05	0.003	65.817	81.8	13.991	29.461	116645504	116645995
PAX5_peak_4951	57.82	-1.249	-3.209	0.001	0.018	94.144	68.167	31.481	37.496	115420792	115421430
PAX5_peak_4980	80.22	-0.967	-2.937	0.003	0.034	109.14	103.01	50.719	58.029	116261760	116262315
PAX5_peak_4935	78.21	-1.018	-2.879	0.004	0.038	128.3	80.285	51.594	52.673	114559170	114559811
PAX5_peak_4962	55.66	-1.098	-2.689	0.007	0.056	94.977	56.048	32.355	39.281	115783152	115783717
PAX5_peak_4959	34.77	-1.226	-2.557	0.01	0.073	47.488	49.989	16.615	24.997	115731781	115732333
PAX5_peak_4994	20.89	-1.335	-2.214	0.026	0.135	32.492	27.266	13.991	9.82	116642334	116642774
PAX5_peak_5004	26.69	-1.164	-2.174	0.029	0.143	31.659	42.415	15.74	16.962	116893825	116894359
PAX5_peak_4997	23.16	-1.215	-2.074	0.038	0.169	41.656	22.722	13.991	14.284	116686368	116686763
PAX5_peak_4985	39.07	-0.908	-2.014	0.044	0.185	59.152	42.415	28.857	25.89	116403612	116404154
PAX5_peak_4946	27.56	-0.902	-1.696	0.089	0.289	44.156	27.266	20.987	17.855	115232421	115232908
PAX5_peak_4965	51.75	-0.66	-1.686	0.091	0.293	67.484	59.078	37.602	42.852	115848009	115848418
PAX5_peak_4961	33.38	-0.608	-1.255	0.209	0.472	42.49	37.87	32.355	20.533	115751308	115751839
PAX5_peak_4977	17.15	0.836	1.219	0.222	0.491	12.497	12.118	30.606	13.391	116229108	116229562
PAX5_peak_5010	31.94	0.614	1.219	0.222	0.491	27.493	22.722	48.096	29.461	117046284	117046584
PAX5_peak_4967	20.07	0.741	1.114	0.265	0.539	21.661	7.5741	34.104	16.962	115884627	115885044
PAX5_peak_4992	23.59	-0.546	-0.959	0.337	0.611	35.824	19.692	19.238	19.64	116550165	116550638
PAX5_peak_4973	18.22	-0.585	-0.935	0.349	0.623	22.494	21.207	12.242	16.962	116042685	116042985

PAX5_peak_4958	22.84	-0.53	-0.929	0.352	0.626	22.494	31.811	20.987	16.069	115707103	115707635
PAX5_peak_4953	15.15	-0.583	-0.826	0.408	0.671	12.497	24.237	9.619	14.284	115511658	115512198
PAX5_peak_5016	20.48	-0.497	-0.819	0.412	0.675	30.826	16.663	18.364	16.069	117238104	117238447
PAX5_peak_4976	43.69	-0.349	-0.796	0.425	0.687	36.658	62.107	36.728	39.281	116219764	116220117
PAX5_peak_4933	74.42	-0.249	-0.739	0.459	0.713	79.981	81.8	74.331	61.6	114466306	114466863
PAX5_peak_4998	19.18	0.423	0.689	0.49	0.736	17.495	15.148	25.359	18.748	116745638	116746002
PAX5_peak_5003	58.35	0.265	0.689	0.491	0.736	43.323	63.622	57.7158	68.742	116885865	116886170
PAX5_peak_4999	23.53	-0.388	-0.669	0.503	0.744	23.327	30.296	27.108	13.391	116757190	116757574
PAX5_peak_4966	47.54	0.258	0.628	0.53	0.765	39.99	46.959	58.59	44.638	115853579	115854300
PAX5_peak_4987	31.18	0.282	0.574	0.565	0.787	31.659	24.237	37.602	31.246	116431681	116432096
PAX5_peak_5014	15.78	0.343	0.514	0.606	0.813	14.163	13.633	18.364	16.962	117190501	117190981
PAX5_peak_5007	22.97	0.287	0.476	0.634	0.83	17.495	24.237	34.979	15.176	116929919	116930219
PAX5_peak_4934	34.32	0.205	0.402	0.687	0.86	41.656	21.207	27.108	47.316	114552752	114553205
PAX5_peak_5000	29.87	-0.11	-0.219	0.826	0.932	31.659	30.296	23.611	33.924	116765364	116765786
PAX5_peak_4990	21.76	0.1	0.176	0.859	0.946	20.828	21.207	23.611	21.426	116496004	116496536

ATAC-seq Igh

Gene	base Mean	log2Fold Change	stat	pvalue	padj	RAG2ko rep 1	RAG2ko rep 2	IL7RxRAG rep 1	IL7RxRAG rep 2
12_116500351..116501400	58.65	-1.607	-3.907	9.34E-05	0.002	104.975	71.196	21.862	36.603
12_116645251..116646300	47.76	-1.757	-3.892	9.93E-05	0.002	65.817	81.8	13.991	29.461
12_115420951..115421400	57.82	-1.249	-3.209	0.001	0.017	94.144	68.167	31.481	37.496
12_116261701..116262450	80.22	-0.967	-2.937	0.003	0.033	109.141	103.007	50.719	58.029
12_114559051..114559800	78.21	-1.018	-2.879	0.004	0.038	128.303	80.285	51.594	52.673
12_115783051..115783650	55.66	-1.098	-2.689	0.007	0.056	94.977	56.048	32.355	39.281
12_115731751..115732350	34.77	-1.226	-2.557	0.01	0.072	47.488	49.989	16.615	24.997
12_116893801..116894400	26.69	-1.164	-2.174	0.029	0.143	31.659	42.415	15.74	16.962
12_116403751..116404050	39.07	-0.908	-2.015	0.043	0.184	59.152	42.415	28.857	25.89
12_115847401..115848000	51.75	-0.661	-1.686	0.091	0.293	67.484	59.078	37.602	42.852
12_115751401..115751850	33.31	-0.609	-1.255	0.209	0.477	42.49	37.87	32.355	20.533
12_115825501..115825800	14.5	0.894	1.204	0.228	0.498	5.831	15.148	21.862	15.176
12_115850101..115850700	31.22	0.594	1.176	0.239	0.512	30.826	18.177	41.975	33.924
12_115884601..115885050	20.07	0.741	1.114	0.265	0.538	21.661	7.574	34.104	16.962
12_116550151..116550450	23.59	-0.546	-0.959	0.337	0.611	35.824	19.692	19.238	19.64
12_115707001..115707600	22.84	-0.53	-0.929	0.352	0.626	22.494	31.811	20.987	16.069
12_116219551..116220450	43.69	-0.349	-0.796	0.425	0.689	36.658	62.107	36.728	39.281
12_115617001..115617600	38.55	-0.33	-0.705	0.48	0.728	49.155	36.355	43.724	24.997
12_116885701..116886450	58.35	0.265	0.689	0.49	0.735	43.323	63.622	57.715	68.742
12_116757301..116757600	23.53	-0.389	-0.669	0.503	0.744	23.327	30.296	27.108	13.391
12_115853401..115854450	47.54	0.258	0.628	0.53	0.764	39.99	46.959	58.59	44.638
12_116431651..116432250	31.18	0.282	0.5746	0.565	0.786	31.659	24.237	37.602	31.246
12_116929951..116930250	22.97	0.287	0.476	0.634	0.83	17.495	24.237	34.979	15.176
12_116765401..116765850	29.87	-0.11	-0.219	0.826	0.931	31.659	30.296	23.611	33.924

S Table 3: EBF1, PAX5 and their key targets are differentially expressed and accessible in IL-7R$\alpha^{-/-}$ Rag2$^{-/-}$ relative to Rag2$^{-/-}$ pro-B cells. Related to Figure 4 and Figure S4. **Differentially expressed genes.** RNA-seq raw reads were quantified over all genes, separately for each of two replicates. The adjusted mean for each probe, as well as the log2-fold change and adjusted p-value in the IL-7R$\alpha^{-/-}$ Rag2$^{-/-}$ relative to Rag2$^{-/-}$ samples was calculated by DESeq2. **Pax5 Igh. Pax5 binding sites show changes in accessibility in IL-7R$\alpha^{-/-}$ Rag2$^{-/-}$ relative to Rag2$^{-/-}$ pro-B cells.** ATAC-seq raw reads were quantified over PAX5 binding sites (only PAX5 binding sites over the Igh locus are shown) for each of two replicates. The adjusted mean for each probe, as well as the log2-fold change and adjusted p-value in the IL-7R$\alpha^{-/-}$ Rag2$^{-/-}$ relative to Rag2$^{-/-}$ samples was calculated by DESeq2. **ATAC-seq Igh.** Location of 24 accessible sites in Igh.
