# Supplementary material for: IL-7R signaling activates widespread VH and DH gene usage to drive antibody diversity in bone marrow B cells
Source: Cell Rep. 2021 Jul 13;36(2):109349. doi: 10.1016/j.celrep.2021.109349 (PMC8293627; doi:10.1016/j.celrep.2021.109349)
Supplement: Document S2. Article plus supplemental information [file mmc5.pdf]

Report

Cell Reports

IL-7R signaling activates widespread V_H and D_H gene usage to drive antibody diversity in bone marrow B cells

Graphical abstract

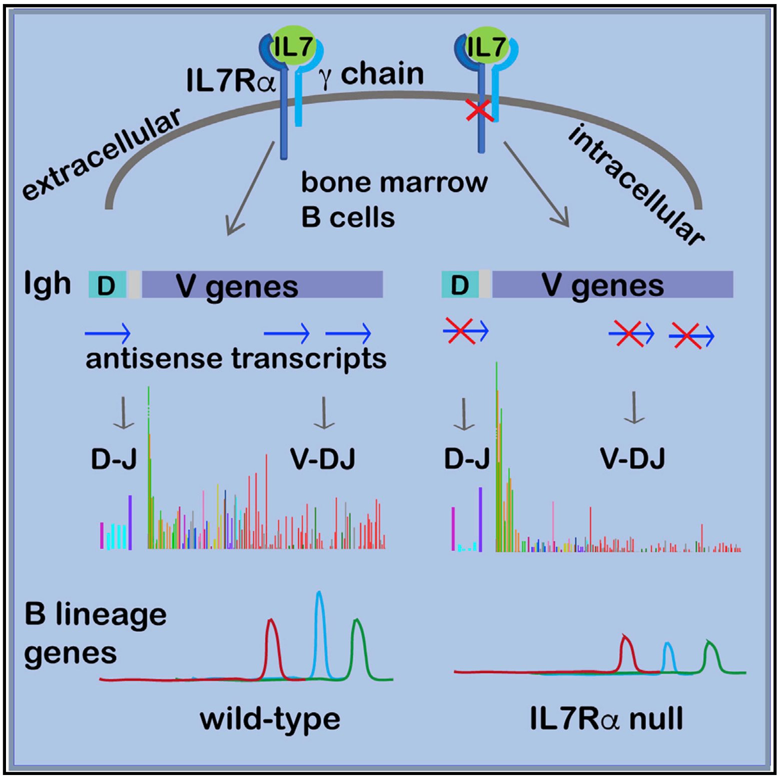

Authors

Amanda Baizan-Edge, Bryony A. Stubbs, Michael J.T. Stubbington, Daniel J. Bolland, Kristina Tabbada, Simon Andrews, Anne E. Corcoran

Correspondence

anne.corcoran@babraham.ac.uk

In brief

Baizan-Edge et al. show that the interleukin-7 receptor drives antibody repertoire diversity. Deletion of the IL-7R impairs usage of most V_H genes and D_H genes in V(D)J recombination of the Igh, causing a severely restricted repertoire. Defects include reduced Igh antisense transcription and diminished expression of B lineage transcription factors.

Highlights

- The IL-7R drives recombination of V_H and D_H genes in the immunoglobulin heavy chain
- Deletion of the IL-7R impairs usage of all except 3′ V_H and flanking D_H genes
- IL-7R loss diminishes large-scale V_H and D_H antisense transcription in the Igh
- IL-7R loss causes reduced expression of B lineage transcription factors and targets

Baizan-Edge et al., 2021, Cell Reports *36*, 109349
July 13, 2021 © 2021 The Authors.
https://doi.org/10.1016/j.celrep.2021.109349

CellPress

Cell Reports

CellPress
OPEN ACCESS

Report

IL-7R signaling activates widespread V_H and D_H gene usage to drive antibody diversity in bone marrow B cells

Amanda Baizan-Edge,[1,4] Bryony A. Stubbs,[1] Michael J.T. Stubbington,[1,5] Daniel J. Bolland,[1,2] Kristina Tabbada,[1,2,6] Simon Andrews,[3] and Anne E. Corcoran[1,2,7,*]

[1]Nuclear Dynamics Programme, Babraham Institute, Babraham Research Campus, Cambridge CB22 3AT, UK
[2]Lymphocyte Signaling and Development Programme, Babraham Institute, Babraham Research Campus, Cambridge CB22 3AT, UK
[3]Bioinformatics Group, Babraham Institute, Babraham Research Campus, Cambridge CB22 3AT, UK
[4]Present address: Centre for Gene Regulation and Expression, School of Life Sciences, University of Dundee, Dow Street, Dundee DD1 5EH, UK
[5]Present address: 10x Genomics, 6230 Stoneridge Mall Road, Pleasanton, CA 94588, USA
[6]Present address: Palaeogenomics, PalaeoBARN, School of Archaeology, University of Oxford, Oxford OX1 3TG, UK
[7]Lead contact
*Correspondence: anne.corcoran@babraham.ac.uk
https://doi.org/10.1016/j.celrep.2021.109349

SUMMARY

Generation of the primary antibody repertoire requires V(D)J recombination of hundreds of gene segments in the immunoglobulin heavy chain (*Igh*) locus. The role of interleukin-7 receptor (IL-7R) signaling in *Igh* recombination has been difficult to partition from its role in B cell survival and proliferation. With a detailed description of the *Igh* repertoire in murine IL-7R$\alpha^{-/-}$ bone marrow B cells, we demonstrate that IL-7R signaling profoundly influences V_H gene selection during V_H-to-DJ_H recombination. We find skewing toward 3′ V_H genes during *de novo* V_H-to-DJ_H recombination more severe than the fetal liver (FL) repertoire and uncover a role for IL-7R signaling in D_H-to-J_H recombination. Transcriptome and accessibility analyses suggest reduced expression of B lineage transcription factors (TFs) and targets and loss of D_H and V_H antisense transcription in IL-7R$\alpha^{-/-}$ B cells. Thus, in addition to its roles in survival and proliferation, IL-7R signaling shapes the *Igh* repertoire by activating underpinning mechanisms.

INTRODUCTION

Interleukin-7 (IL-7) is a critical cytokine for B and T lymphocyte development. It is bound by the IL-7 receptor (IL-7R), composed of the common gamma chain (γc), shared with the IL-2R, and the IL-7-specific IL-7Rα chain (CD127—encoded by *Il7r*), which also pairs with the thymic stromal lymphopoietin receptor (TSLPR), important in fetal liver (FL) B cell survival (Vosshenrich et al., 2003; Rochman et al., 2009). Binding of IL-7 to the IL-7R in pro-B cells activates several signaling pathways, including Janus kinase/signal transducers and activators of transcription (JAK/STAT), phosphoinositide-3 kinase (PI3K)/Akt (Protein kinase B), mitogen-activated protein kinase/extracellular signal-regulated kinase (MAPK/ERK), and PLCγ/PKC/mammalian target of Rapamycin (mTOR), and has been associated with proliferation and survival, B lineage commitment, and *Igh* recombination (Corcoran et al., 1996, 1998; reviewed by Corfe and Paige, 2012; Yu et al., 2017).

IL-7R signaling is essential for B lineage commitment. Its absence in IL-7R$\alpha^{-/-}$ mice impairs early B cell development from the common lymphoid progenitor (CLP) stage, resulting in reduced progenitors and impaired B-lineage potential (Peschon et al., 1994; Miller et al., 2002; Erlandsson et al., 2004; Dias et al., 2005, Medina et al., 2013). This is due in part to failure to activate early B-Cell Factor 1 (EBF1), a crucial transcription factor (TF) for B lineage specification (Kikuchi et al., 2005; Tsapogas et al., 2011; Pongubala et al., 2008; Roessler et al., 2007; Boller and Grosschedl, 2014). Paired box 5 (PAX5), a key TF for B cell commitment (Nutt et al.; 1999; Rumfelt et al., 2006), is also reduced in IL-7R$\alpha^{-/-}$ pro-B cells (Corcoran et al., 1998), but this may be due to reduced EBF1 expression (O'Riordan and Grosschedl 1999; Decker et al., 2009; Lin et al., 2010). However, some cells progress to the pre-pro-B stage (Kikuchi et al., 2005; Peschon et al., 1994; Miller et al., 2002) and fewer to the CD19$^+$ pro-B compartment (Corcoran et al., 1998; Miller et al., 2002).

Although IL-7R$\alpha^{-/-}$ pro-B cells in the bone marrow (BM) undergo *Igh* V_H-DJ_H recombination (Corcoran et al., 1998), their usage of V_H genes is severely restricted, indicating a role of IL-7R signaling in this process. Importantly, this is independent of proliferation: IL-7R$\alpha^{-/-}$ cells expressing an IL-7Rα chain lacking Tyr449, required for PI3K signaling, express a recombined Igμ polypeptide but do not proliferate *in vitro* (Corcoran et al., 1996). Conversely, a chimeric receptor comprising the IL-7Rα extracellular domain and the IL-2Rβ intracellular domain restored proliferation in IL-7R$\alpha^{-/-}$ cells, but not Igμ expression, indicating a non-redundant role for the IL-7R in *Igh* V(D)J recombination.

This is an open access article under the CC BY license (http://creativecommons.org/licenses/by/4.0/).

A diverse antibody repertoire requires inclusion of all available V_H and D_H genes. Large-scale processes, including non-coding transcription and Ig locus contraction, are thought to facilitate accessibility of distal 5′ V_H genes to the recombination center over the DJ region, where the recombination activating gene (RAG) 1 and 2 bind (Ji et al., 2010, Corcoran et al., 1998; Bolland et al., 2004; Yancopoulos and Alt, 1985, Chowdhury and Sen, 2003; Fuxa et al., 2004; Jhunjhunwala et al., 2008; Sayegh et al., 2005; Stubbington and Corcoran, 2013). Nevertheless, V_H genes recombine at widely different frequencies; frequently recombining V_H genes also have one of two local active chromatin states (Bolland et al., 2016).

IL-7R$\alpha^{-/-}$ pro-B cells *in vivo* displayed decreased non-coding transcription and recombination of 5′ V_H genes (Corcoran et al., 1998), prompting the hypothesis that the IL-7R influences *Igh* recombination through increasing accessibility of 5′ V_H genes, supported by studies linking IL-7R signaling and active histone modifications in the *Igh* locus (Chowdhury and Sen, 2001; Johnson et al., 2003; Xu et al., 2008), and suggesting that IL-7R activation of the *Igh* locus is mediated by STAT5 (Bertolino et al., 2005). However, conditional deletion of STAT5 was rescued by the pro-survival factor B-Cell Lymphoma-2 (BCL-2) with no deficiency in 5′ V_H recombination, suggesting that the dominant role of STAT5 was pro-B cell survival (Malin et al., 2010). IL-7R$\alpha^{-/-}$ B cells were only partially rescued and cannot be rescued by Eu-BCL-2 expression (Maraskovsky et al., 1998), indicating that the IL-7R has additional downstream signaling roles. Heterogeneous expression of the IL-7R during the cell cycle both inhibits Rag recombinase expression to prevent DNA breaks during replication, while maintaining *Igh* locus accessibility throughout the cell cycle (Johnson et al., 2012).

Other studies have suggested that loss of IL-7Rα prevents B cell progression beyond the pre-pro-B stage and that B cells in the BM originate from the FL (Kikuchi et al., 2005; Peschon et al., 1994; Miller et al., 2002; Carvalho et al., 2001), supported by similarity in V_H repertoire between IL-7R$\alpha^{-/-}$ and FL B cells (Corcoran et al., 1998; Jeong and Teale, 1988; Yancopoulos et al., 1988). Definitive conclusions have been hampered by incomplete knowledge of the *Igh* locus, provided later (Johnston et al., 2006), and low-resolution *Igh* repertoire assays.

With next-generation sequencing (NGS) enabling more comprehensive analysis of *Igh* repertoires, we have revisited the IL-7R$\alpha^{-/-}$ mouse (Peschon et al., 1994) to address the uncertainties above, which confound a complete picture of the role of the IL-7R in B cell development. Using VDJ sequencing (VDJ-seq) (Bolland et al., 2016), a DNA-based NGS technique, we have fully characterized the *Igh* DJ_H and VDJ_H repertoires in IL-7R$\alpha^{-/-}$ BM B cells. Widespread use of gene segments in both D_H-J_H and V_H-D_H recombination was severely impaired by loss of IL-7R signaling.

Importantly, IL-7R$\alpha^{-/-}$ BM B cells are not derived from the FL. Junctions between V_H, D_H, and J_H gene segments in IL-7R$\alpha^{-/-}$ BM B cells are much more variable than FL B cell sequences, indicating that IL-7R$\alpha^{-/-}$ BM pro-B cells undergo *de novo* V(D)J recombination. Furthermore, they have a much more severe reduction in repertoire diversity than FL. Transcriptome analysis reveals loss of large-scale antisense intergenic transcripts in both D_H and V_H regions and reduced expression of key transcription factors required for *Igh* recombination, including EBF1 and Pax5. Thus, IL-7R signaling promotes *Igh* repertoire diversity in BM pro-B cells by activating mechanisms that enable large-scale access to V_H and D_H genes.

RESULTS

Usage of V and D genes in *Igh* recombination is severely skewed in pro-B cells lacking the IL-7Rα chain

VDJ-seq was performed on two IL-7R$\alpha^{-/-}$ BM and two wild-type (WT) FL pro-B cell samples and compared with two WT BM datasets we previously generated (GEO: GSE80155; Bolland et al., 2016). Replicate libraries for all genotypes were highly correlated, indicating VDJ-seq is highly reproducible (Figure S1). Although IL-7R$\alpha^{-/-}$ libraries were generated with approximately 6-fold fewer reads (Figure S2), the ratio of VDJ_H to DJ_H recombined products was similar to WT, indicating that dynamic progression through first D_H to J_H, followed by V_H to DJ_H recombination, was not slowed (Figure S3). A binomial test was applied to determine genes with significantly greater read counts than expected by chance, considered to be actively recombining (Bolland et al., 2016). Consistent with a requirement for IL-7R signaling to enable usage of V_H genes in recombination, fewer V_H genes passed the binomial test in IL-7R$\alpha^{-/-}$ (84 genes) relative to WT pro-B cells (128 genes). All but three were within the WT group (Table S1).

To visualize V_H gene recombination frequencies and compare between genotypes, V_H gene quantifications were normalized to the total number of reads over all V_H genes for each genotype (Figure 1A). A much higher proportion of sequences mapped to the most 3′ V_H genes in IL-7R$\alpha^{-/-}$ than in the WT repertoire (Figure 1B). Notably, the first five V_H genes comprised 45% of the total repertoire, compared with 20% in WT. Of the 44 V_H genes that recombine in WT, but not in IL-7R$\alpha^{-/-}$ BM, the vast majority were at the 5′ end of the V region, including several that normally recombine at high frequency (J558.16.106, J558.26.116, and J558.67.166).

The 195 V_H genes segregate into 16 families in 3 clans (Johnston et al., 2006). Their diverged evolutionary origins correlate with differential TF binding and chromatin state around V_H genes (Bolland et al., 2016). Because accessibility, TF expression, and chromatin state have been linked to IL-7R signaling, we investigated the relationship between recombination frequency and V_H gene clan or chromatin state in IL-7R$^{-/-}$ pro-B cells. V_H genes in clan 1 recombine at lower frequency in the IL-7R$\alpha^{-/-}$, although clan 2 and 3 V_H genes recombine at similar frequencies to WT (Figure 1C). When the data are broken down to V_H gene families, a more nuanced picture emerges. Within clan 2 and 3, 7183 and Q52, the two largest and most 3′ V_H families, recombine more frequently in IL-7R$\alpha^{-/-}$. However, several of the smaller families in the middle region recombine less frequently (Figure S3). The enhancer state V_H genes (including clan 1 and the distal 3609 family from clan 2) were significantly less frequently recombined relative to the architectural state V_H genes (clan 2, except 3609, and clan 3; Figure 1D). However, some architectural state V_H families also recombined less frequently. Thus, loss of IL-7R impacts on V_H genes in the enhancer state (distal and middle

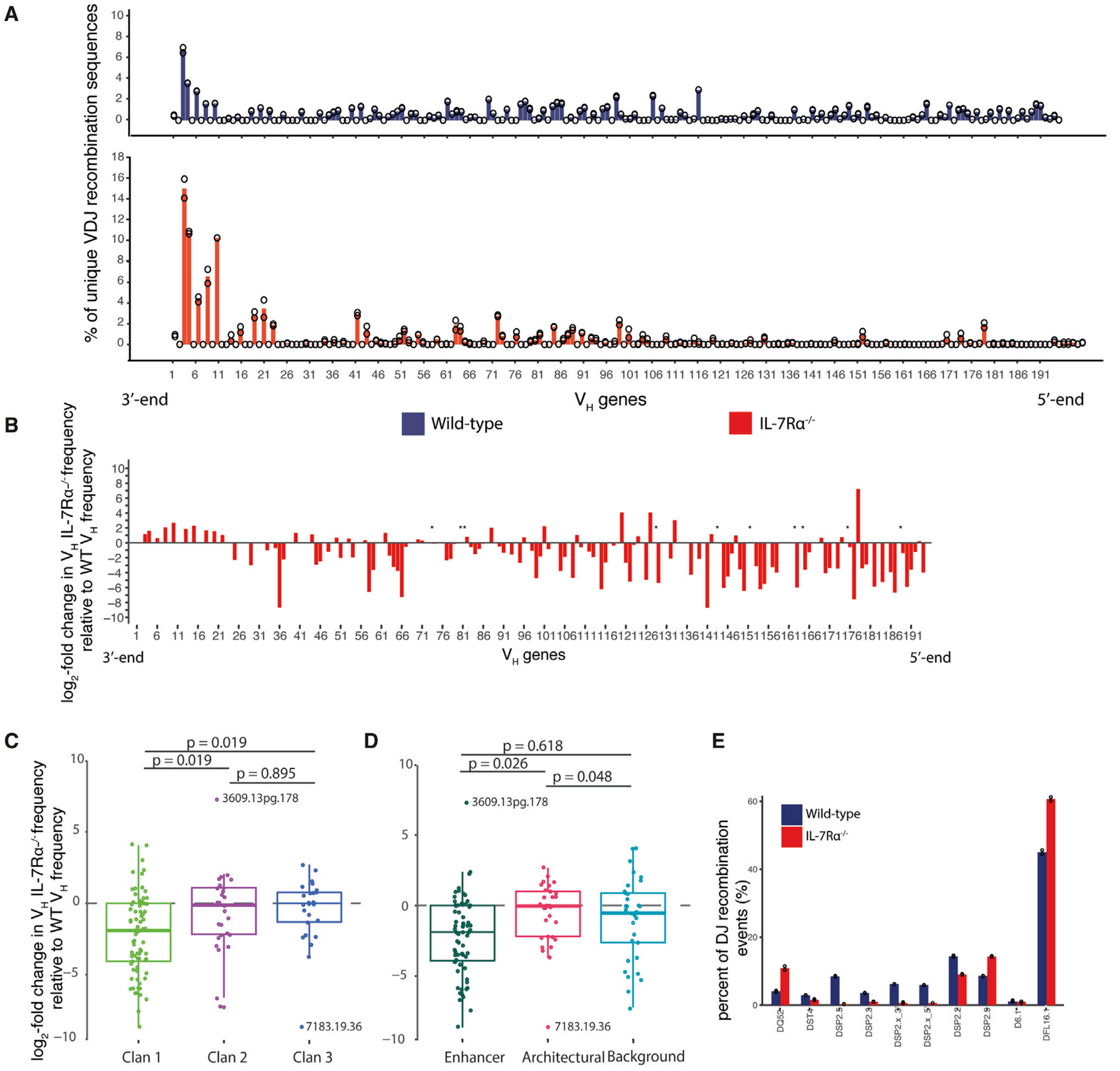

Figure 1. IL-7Rα$^{-/-}$ pro-B cells have impaired V_H and D_H repertoires

(A) Recombination frequencies of 195 V_H genes measured by VDJ-seq. WT BM pro-B cells (blue) and IL-7Rα^{-}/$^{-}$ (red) are shown. Two biological replicates are shown as open circles (bar height represents average). Reads for each V_H gene are shown as percentage of total reads quantified. V_H gene number legend is shown in Table S1.

(B) The mean of each V_H gene was divided with the WT mean followed by log2 transformation. Only genes recombining in either genotype are shown. * represents V_H genes with value 0 in IL-7Rα^{-}/$^{-}$ (only in WT). V_H gene raw read counts and recombining/non-recombining classification are shown in Table S1.

(C and D) Log2 values for each gene in graph B (excluding those marked by *) were grouped by (C) evolutionary origin: clan 1 (n = 78), clan 2 (n = 27), and clan 3 (n = 26); ANOVA (degrees freedom [Df] = 2; F-value = 5.39; p = 0.005) and (D) chromatin state: enhancer (n = 68), architectural (n = 30), and background (n = 33); ANOVA (Df = 2; F-value = 4.54; p = 0.012).

(E) Reads for each D_H gene as percentage of total reads quantified for two biological replicates of WT (blue) and IL-7Rα$^{-/-}$ (red) pro-B cells.

genes) and on several middle region families in the architectural state. This distribution also applies to the clans: loss of IL-7R reduces recombination of clan 1 (mostly distal V genes) as well as the middle genes from clans 2 and 3. Importantly, this suggests that the IL-7R does not influence either clans or local chromatin states selectively but rather linear positioning in the *Igh* V region, i.e., loss of IL-7R impairs recombination of middle and 5′ V genes in the *Igh* locus.

Actively recombining D_H genes were also identified by binomial test. VDJ-seq revealed profound changes in individual D_H usage in IL-7R$\alpha^{-/-}$ pro-B cells. Several centrally positioned DSP gene segments (DSP2×5′, DSP2×3′, DSP2.3, and DSP2.5) do not recombine in IL-7R$\alpha^{-/-}$ pro-B cells (Figure 1E). Conversely, relative recombination frequencies of the most 3′ D_H gene, DQ52; the most 5′, DFL16.1; and its adjacent DSP2.9 were increased in IL-7R$\alpha^{-/-}$ cells.

IL-7R$\alpha^{-/-}$ pro-B cells do not originate from the FL

We investigated whether recombination events in IL-7R$\alpha^{-/-}$ BM were comparable to B cells derived from FL, rather than *de novo* in the BM. VDJ-seq data from WT embryonic day 15.5 pro-B cells were compared with IL-7R$\alpha^{-/-}$ and WT BM. Notably, the ratio of DJ to VDJ recombinants in FL was 12:1, in contrast to WT and IL-7R$\alpha^{-/-}$ BM ratios of approximately 2:1 and 1:1, respectively (Figure S3). Consistent with previous reports, the FL V_H gene repertoire exhibited a 3′ bias relative to WT, including more frequent use of the most recombined V_H gene, V_H-81X (11% compared with 7% for WT BM; Figures 2A and S2C). However, IL-7R$\alpha^{-/-}$ cells had a more pronounced phenotype, with V_H-81X comprising 14% of VDJ recombined sequences. Additionally, many 5′ V_H genes recombined less frequently than in FL pro-B cells (Figure 2A). V_H usage within *Igh* V_H gene family sub-domains (Figure 2B) is distributed evenly across the locus in WT but is somewhat biased toward the D-proximal 3′ gene families in FL B cells. However, this shift is mild for all but the central and distal J558 genes (Figure 2C). The IL-7R$\alpha^{-/-}$ repertoire is also biased toward the 7183/Q52 V_H family but much more severely than FL B cells. In contrast to FL, this increase in 7183/Q52 V_H gene usage was mirrored by a decrease in usage for every other family except the small J606 family. Thus, recombination in IL-7R$\alpha^{-/-}$ cells is markedly more biased toward the 3′ V_H genes than FL, suggesting IL-7R$\alpha^{-/-}$ BM pro-B cells do not simply originate from FL precursors.

To distinguish whether VDJ_H sequences in IL-7R$\alpha^{-/-}$ BM cells are derived from FL or adult BM, we analyzed VDJ-seq libraries with IMGT to compare non-templated (N-) incorporations within VD_H and DJ_H junctions. Terminal deoxynucleotide transferase (TdT), which inserts N-nucleotides, is absent in FL and first expressed in pro-B cells in the BM (Feeney, 1990; Li et al., 1993). Accordingly, we identified very few N-additions in FL junctions; only 25% and 15% had more than one incorporation in the VD and DJ junction, respectively. In contrast, around 80% of VD and DJ junctions in WT BM had N-additions (Figures 2D and 2E). 60% of VD and DJ joins from IL-7R$\alpha^{-/-}$ cells also had N-additions. IL-7R$\alpha^{-/-}$ and WT sequences also had a similar distribution, including up to 10 additions (Figures 2D and 2E). Together, these data demonstrate that IL-7R$\alpha^{-/-}$ pro-B cells undergo V(D)J recombination *de novo* in the BM, but loss of the IL-7Rα severely restricts usage of V_H and D_H genes in the formation of the primary *Igh* repertoire.

IL-7R signaling does not influence local V gene chromatin state

We next investigated how IL-7R signaling may regulate V(D)J recombination. Reduction in recombination of 5′ V_H genes in IL-7R$\alpha^{-/-}$ BM, together with normal DJ/VDJ ratios, suggests that signaling through IL-7R is specifically required for 5′ V_H gene recombination. We first asked whether altered Recombination Signal Sequence (RSS) accessibility could account for this reduced recombination, because the local enhancer chromatin state is predominantly associated with 5′ V_H genes (Bolland et al., 2016). We performed ATAC-seq to identify accessible DNA in a chromatin context (Pulivarthy et al., 2016). We performed these experiments in Rag recombinase-deficient models, which cannot recombine Ig loci, thereby enabling analysis of the intact, poised *Igh* locus and avoiding interference from loss of sequence, or elevated V_H gene expression, due to recombination. We compared Rag2$^{-/-}$ pro-B cells (which express the endogenous IL-7R) with IL-7R$\alpha^{-/-}$ × Rag2$^{-/-}$ (referred to as IL-7Rα/Rag2$^{-/-}$) BM. Duplicate libraries for both genotypes were highly correlated, indicating the data are highly reproducible (Figure S1).

In Rag2$^{-/-}$ pro-B cells, V_H RSSs coincided with a peak of accessibility (Figure 3A). V_H RSSs in IL-7Rα/Rag2$^{-/-}$ cells had a similar highly accessible profile, suggesting IL-7R signaling does not activate local accessibility over V_H RSSs. The Igκ light chain V gene (Vκ) RSSs were used as a negative control because the Igκ locus does not become fully activated until the pre-B stage (Matheson et al., 2017). Accordingly, Vκ RSSs were less accessible than V_H RSSs (Figures 3A and 3B). Notably, this pattern was similar in Rag2$^{-/-}$ and IL-7Rα/Rag2$^{-/-}$ pro-B cells, suggesting that Igκ Vκ genes are not activated prematurely in the absence of the IL-7Rα.

We divided the RSSs into clans and chromatin states to test whether these groups showed different accessibility patterns in IL-7Rα/Rag2$^{-/-}$. There was no significant difference between clans (Figure 3C). RSS accessibility in both enhancer and architectural groups was significantly reduced compared to the background state V_H genes, which showed a small increase in RSS accessibility relative to WT (Figure 3D). Together, these data indicate little difference in local accessibility at recombining V_H genes in the absence of the IL-7Rα.

Igh antisense intergenic transcription, but not V_H genic transcription, is impaired in IL-7R$\alpha^{-/-}$ pro-B cells

Non-coding transcription has been proposed to promote chromatin accessibility to facilitate *Igh* V_H gene recombination. The *Igh* V_H region has short non-coding genic sense transcripts at V_H promoters (Yancopoulos and Alt, 1985; Corcoran et al., 1998) and long intergenic antisense transcripts (Bolland et al., 2004, 2016; Verma-Gaur et al., 2012). To investigate changes in the absence of IL-7R signaling, we performed RNA-seq (Figures S1G–S1I). This revealed that there is generally little V_H genic transcription over the 3′ V genes; 31 of the 39 most D-proximal V genes showed no transcription in Rag2$^{-/-}$ pro-B cells. Very few non-coding V_H genic transcripts were differentially expressed between IL-7Rα/Rag2$^{-/-}$ and Rag2$^{-/-}$ cells (Table S2). Notable exceptions included all four members of the middle region SM7 family, highly abundant in Rag2$^{-/-}$ but almost completely absent in IL-7Rα/Rag2$^{-/-}$ cells. Conversely, two of the most 5′ V_H genes, J558.88.194 and J558.87.193, not transcribed in Rag2$^{-/-}$ pro-B cells, are highly expressed in IL-7Rα/Rag2$^{-/-}$ pro-B cells (Figure 3E). Long intergenic antisense non-coding transcripts in the V_H (Pax5-activated intergenic repeat 4 [PAIR4], PAIR6, J558, and J606) and DJ_H regions (Iμ, μ0, and

CellPress
OPEN ACCESS

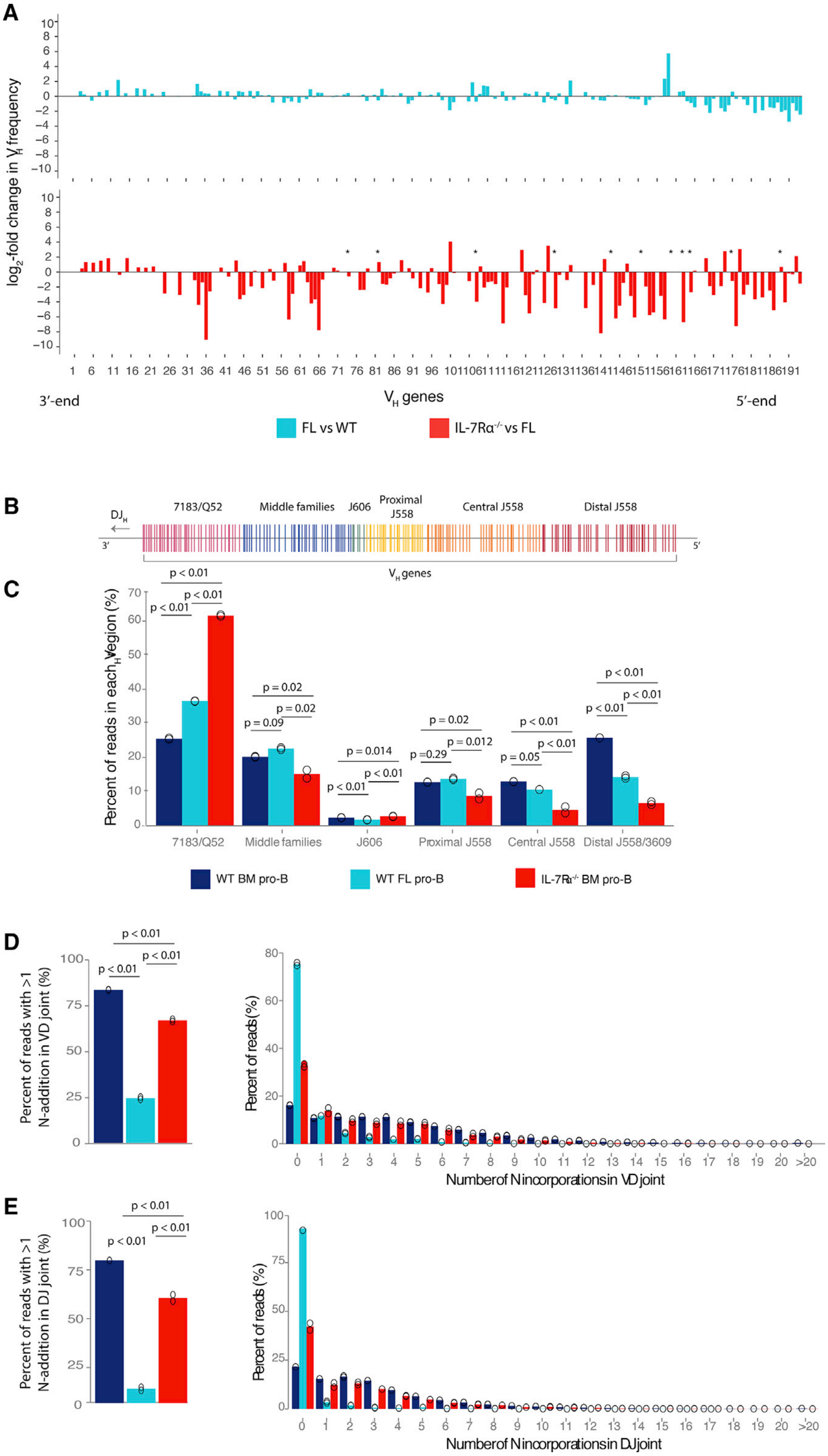

Figure 2. V$_H$ gene use in FL pro-B cells is less restricted than IL-7Rα$^{-/-}$, and VDJ sequences in IL-7Rα$^{-/-}$ and WT show similar N-incorporations

(A) Average of two WT, two FL, and two IL-7Rα$^{-/-}$ biological replicates calculated for each V$_H$ gene. To display changes between WT and FL frequencies, V$_H$ frequencies for FL were divided with the WT mean value and log2 transformed (light blue). Only genes active in either genotype are shown. FL and IL-7Rα$^{-/-}$ frequencies are compared (red). * represents V$_H$ genes with value 0 in IL-7Rα$^{-/-}$ replicates.

(B) Representation of all V$_H$ gene segments, colored by family domains.

(C) Quantified VDJ-seq reads over each V$_H$ gene merged for each family domain and calculated as a percent of total quantified reads for WT (dark blue) and IL-7Rα$^{-/-}$ (red) BM pro-B cells and wild-type FL pro-B cells (light blue). Each open circle represents a biological replicate (n = 2). 7185/Q52, ANOVA (Df = 2; F-value = 11,961; p = 0.01); middle families, ANOVA (Df = 2; F-value = 29.4; p = 0.01); J606, ANOVA (Df = 2; F-value = 93.14; p = 0.002); proximal J558, ANOVA (Df = 2; F-value = 26.70; p = 0.012); central J558, ANOVA (Df = 2; F-value = 67.82; p = 0.003); distal J558, ANOVA (Df = 2; F-value = 917.6; p < 0.001).

(D and E) VDJ-seq libraries analyzed with IMGT to determine number of nucleotides inserted into junctions during VDJ recombination: (D) junction between V$_H$ and D$_H$ (ANOVA [Df = 2; F-value = 3,578.5; p < 0.001]) and (E) junction between D$_H$ and J$_H$ gene segments (ANOVA [Df = 2; F-value = 1,037.4; p < 0.001]) of WT (dark blue) and IL-7Rα$^{-/-}$ (red) BM and wild-type FL (light blue) recombination events. Number of sequences with more than 1 N-addition (left) and distribution of sequences with N-additions (right) is shown as percent of all mapped VDJ-seq sequences.

D$_H$-antisense) were also analyzed. Although the RNA-seq libraries were not strand specific, these known transcripts are easily distinguished from the much less frequently transcribed genic transcripts in Rag2$^{-/-}$ pro-B cells (Figure 3E). Strikingly, antisense transcription throughout the V$_H$ region was almost completely absent in IL-7Rα/Rag2$^{-/-}$ pro-B cells. DESeq2 revealed significant reduction in all V$_H$ antisense transcripts tested (Table S2). Furthermore, although sense transcription over the J$_H$ (μ0) or the C$_H$ (Iμ) transcript regions was relatively unchanged (Table S2), D$_H$ antisense transcription was almost completely lost over the entire DJ$_H$ region in IL-7Rα/Rag2$^{-/-}$ cells (Figure 3F).

EBF1, PAX5, and other key B-cell-lineage-specifying genes are misregulated in IL-7Rα$^{-/-}$ pro-B cells

We next examined genome-wide alterations in gene expression in the absence of the IL-7R (Figure S4). Gene set enrichment analysis (GSEA) confirmed the essential role of IL-7R signaling in cell cycle and clonal expansion as genes related to E2F, G2M

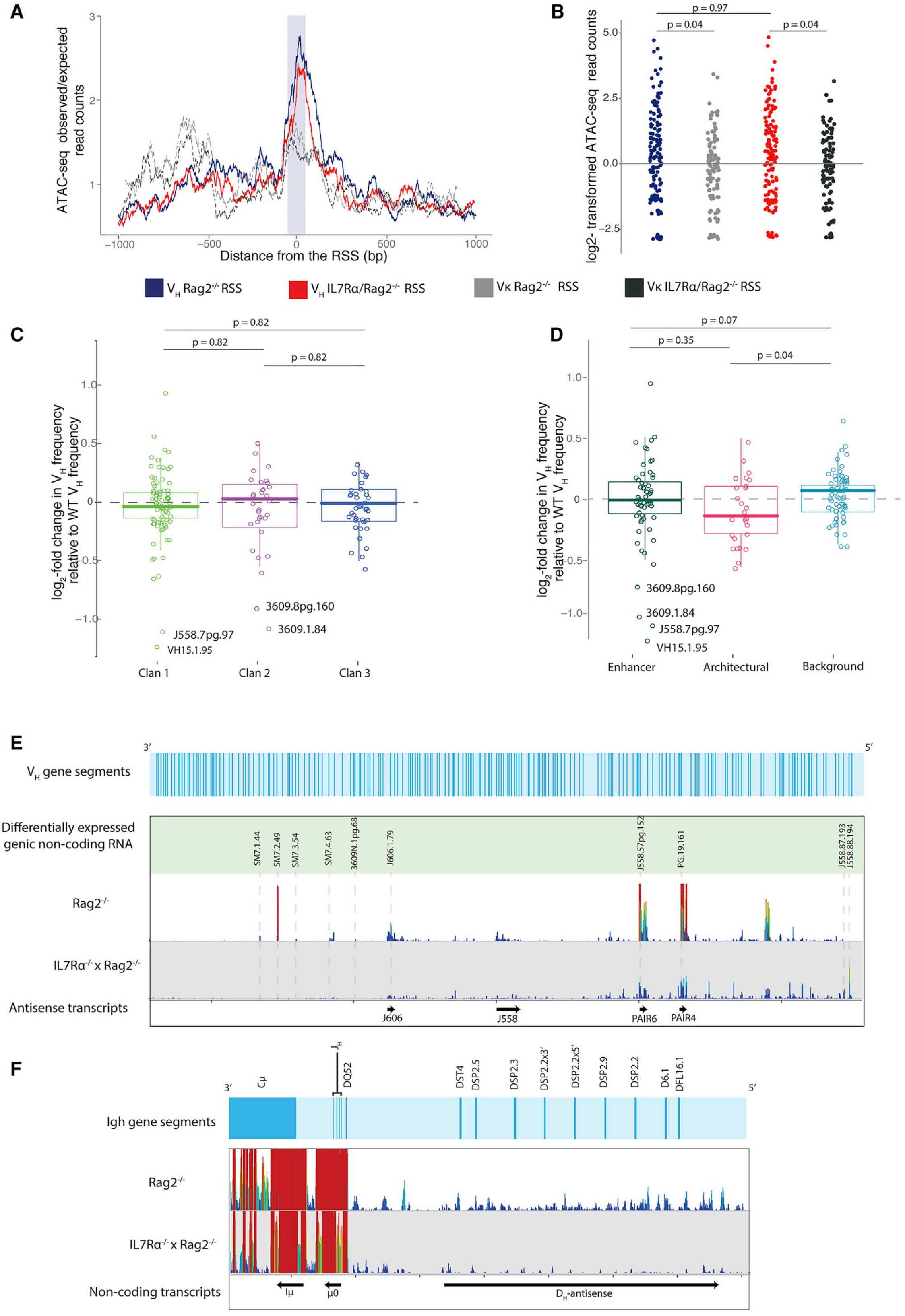

(legend on next page)

checkpoint, and MYC were downregulated in the IL-7Rα/Rag2$^{-/-}$ cells (Figures S4B–S4D). Consistent with previous reports, expression of both *Ebf1* and *Pax5* was substantially reduced in IL-7Rα/Rag2$^{-/-}$ pro-B cells (Table S3). Importantly, several key B-lineage genes regulated by *Ebf1* and *Pax5* had reduced transcription levels in IL-7Rα/Rag2$^{-/-}$ cells, including *Foxo1*, *Rag1*, and *Cd79a* (coding for Igα, pre-B cell receptor complex), downregulated in EBF1-deficient cells (Györy et al., 2012). PAX5-activated genes, including *Smarca4* (encoding BRG1) and *Lef1*, were also decreased. Conversely, FLT3R (*Flt3*), downregulated in pro-B cells by PAX5 (Pridans et al., 2008), was upregulated in IL-7Rα/Rag2$^{-/-}$ pro-B cells. GSEA demonstrated that, overall, genes with PAX5 binding sites at their promoters were depleted in IL-7Rα/Rag2$^{-/-}$ pro-B cells (Figure S4E). However, some B-cell-specific genes regulated by PAX5 and EBF1 were expressed normally: *Irf4* (direct target of both), *Myb*, and *Pdcd1* (activated and repressed by EBF1) showed no significant transcriptional changes in IL-7Rα/Rag2$^{-/-}$ cells. Indeed, *Irf8* and *Ikzf3* (Aiolos), activated by both, were more highly expressed in IL-7Rα/Rag2$^{-/-}$ cells. Together, these results suggest that EBF1 and PAX5 function is mis-regulated in IL-7Rα$^{-/-}$ cells.

To determine whether IL-7R signaling additionally influences the binding pattern of these and other TFs, we compared accessible hypersensitivity sites with ATAC-seq, using the Model-based Analysis of ChIP-Seq (MACS) caller (Zhang et al., 2008) function in Seqmonk. We divided the sites into two groups: those that had fewer reads (less accessible) or more reads (more accessible) in IL-7Rα/Rag2$^{-/-}$ than Rag2$^{-/-}$ cells. We used hypergeometric optimization of motif enrichment (HOMER) to identify TF motifs within these sites. This allowed us to infer TFs that bind less often (less accessible sites) in IL-7Rα/Rag2$^{-/-}$ relative to Rag2$^{-/-}$ and vice versa. EBF1 bound less often in IL-7Rα/Rag2$^{-/-}$ cells, correlating with reduction in its expression and that of its target genes. The PAX5 motif was not found in HOMER. We infer that the PAX8 motif, with a similar binding pattern, is PAX5, because PAX8 is not expressed in B cells. Again, we found reduced representation of this motif in ATAC-seq-accessible sites in IL-7Rα/Rag2$^{-/-}$ cells (Figure 4A).

To further interrogate the differences in PAX5 and EBF1 binding in the *Igh* locus, we examined MACS peaks in published PAX5 (GSM932924) and EBF1 (GSM876622 and GSM876623) chromatin immunoprecipitation (ChIP) data in Rag$^{-/-}$ pro-B cells. We quantified ATAC-seq reads over these sites to infer TF binding differences between IL-7Rα/Rag2$^{-/-}$ and Rag2$^{-/-}$ cells. Of 24 accessible sites in the *Igh* locus (not counting RSSs), all overlapped with one of the 34 PAX5 binding sites. Five showed a marked decrease in accessibility in IL-7Rα/Rag2$^{-/-}$ cells (Table S3). Importantly, two of these sites correspond to the PAIR4 and PAIR6 PAX5 binding sites (Figure S4I), providing a mechanism for the loss of non-coding transcription above. We only detected three EBF1 binding sites in the *Igh* locus, with no difference in accessibility. However, quantification of ATAC-seq reads over 2,896 EBF1 binding sites genome-wide revealed 643 significantly differentially enriched sites. 630 had lower accessibility in IL-7Rα/Rag2$^{-/-}$ cells (Table S4). In the HOMER analysis, we also detected reduced accessibility at motifs of other important TFs involved in B cell specification, including E2A. Reduced specification was reflected in TF motifs at more accessible sites in IL-7Rα/Rag2$^{-/-}$ pro-B cells, which included T cell development motifs (GATA family TFs) and early B cell priming TFs, including PU.1, MYB, and RUNX (Figure 4B). Furthermore, GSEA analysis showed that genes with binding sites for GATA, LIM domain only 2 (LMO2), and NFE2 (TFs in T- and erythroid development) were enriched in IL-7Rα/Rag2$^{-/-}$ pro-B cells (Figures S4F–S4H). Overall, the pattern of TF motif accessibility and target gene alteration suggests that the IL-7Rα enforces commitment to the B cell lineage.

DISCUSSION

Here, we asked whether and how the IL-7R plays a role in *de novo* immunoglobulin gene recombination in BM B cells. As previously reported (Jeong and Teale, 1988; Yancopoulos et al., 1988), we found that FL pro-B cells had a bias toward usage of 3′ V_H genes. However, the FL B cell antibody repertoire was far less restricted than previously thought, suggesting the current model of B cell ontogeny, in which complex antibody repertoires do not develop until after birth warrants revisiting (Siegrist and Aspinall 2009). It will be important to investigate which mechanisms that underpin adult *Igh* repertoire formation are already in place in FL, including long-range looping and local V gene activation, dependent on CCCTC-binding factor (CTCF) and PAX5 (Gerasimova et al., 2015; Bolland et al., 2016; Jain et al., 2018). Because PAX5 is essential for FL *Igh* recombination (Nutt et al., 1997), it may play similar roles in FL B cells.

Our unprecedented depth of analysis of IL-7Rα$^{-/-}$ VDJ$_H$ and DJ$_H$ sequences demonstrates that BM pro-B cells lacking the

Figure 3. IL-7Rα/Rag2$^{-/-}$ pro-B cells show no significant changes in RSS accessibility but striking loss of non-coding transcription over the *Igh* locus

(A) Accessibility over the V_H and Vκ RSSs in Rag2$^{-/-}$ and IL-7Rα/Rag2$^{-/-}$ pro-B cells. Accessibility tracks over a 1,000-bp region centered on the RSS. ATAC-seq reads were quantified over each bp. Each track is an average for all Rag2$^{-/-}$ V_H (blue) and Vκ (dotted gray) RSSs and IL-7Rα/Rag2$^{-/-}$ V_H RSSs (red) and Vκ (dotted black). Purple area represents the RSS.

(B) ATAC-seq reads over a 50-bp region over V_H (n = 195) and Vκ (n = 162) RSS for Rag2$^{-/-}$ and IL-7Rα/Rag2$^{-/-}$. ANOVA (Df = 3; F-value = 3.68; p = 0.012).

(C and D) Differential expression for each RSS calculated by DESeq2 was grouped by (C) evolutionary origin: clan 1 (n = 78), clan 2 (n = 27), and clan 3 (n = 26); ANOVA (Df = 2; F-value = 0.27; p = 0.77) and (D) chromatin state: enhancer (n = 68), architectural (n = 30), and background (n = 33); Kruskal-Wallis test (Df = 2; chi-square = 7.51; p = 0.023) plus pairwise Wilcox test (adjusted by Benjamini and Hochberg method) to calculate p value.

(E and F) RNA-seq reads for Rag2$^{-/-}$ and IL-7Rα/Rag2$^{-/-}$ were quantified per 60-bp bins along the *Igh* locus and normalized by rpm. Height and color of bars represent number of reads over each probe: high red bars have more reads than short blue bars. Each track was generated from average of two biological replicate RNA-seq libraries.

(E) Transcription over the V_H region. Top light blue track: location of all V_H genes is shown. Transcription over ten significantly differentially expressed V_H genes (Table S2) in green track (gray dotted line marks their location) is shown; locations of antisense intragenic non-coding transcripts are shown as black arrows.

(F) Transcription over D_H, J_H and C_H regions. Gene locations are shown in light blue track. Intergenic non-coding transcripts, black arrows.

A

Rank	Motif	TF	P-value	% of Targets Sequences	% of Background Sequences
2	GTCCCCAGGGGA	EBF1	1.00E-04	27.83%	13.31%
8	AAACAGCTGC	E2A	1.00E-03	33.04%	19.23%
10	GTCATGCCTGACTGC	Pax8	1.00E-02	10.43%	3.66%
13	ATATGCAAAT	Oct2	1.00E-02	6.09%	1.52%

B

Rank	Motif	TF	P-value	% of Targets Sequences	% of Background Sequences
1	GCAGATAAGA	GATA4	1.00E-09	33.67%	9.84%
4	CAGATAAGGA	GATA1	1.00E-07	22.45%	5.53%
5	AGATAACA	GATA3	1.00E-07	38.78%	15.56%
8	TCCTTATCTC	GATA2	1.00E-06	22.45%	6.35%
11	CAAACCACAG	RUNX	1.00E-06	24.49%	8.13%
16	AAACCACAAA	RUNX1	1.00E-04	27.55%	11.89%
18	AAAACCACAAAC	RUNX2	1.00E-04	23.47%	9.90%
19	AGAGGAAGTG	PU.1	1.00E-04	15.31%	4.82%
24	GGCGGTTA	MYB	1.00E-02	31.63%	19.16%

Figure 4. TF motif analysis at genomic sites of altered accessibility

Peaks identified from ATAC-seq by MACS peak calling from two biological replicates.

(A) Sites less accessible in IL-7Rα/Rag2$^{-/-}$ analyzed using DESeq2. TF motif enrichment using HOMER is shown. Relevant significantly enriched motifs are shown in order of significance (rank indicated their position in the list).

(B) The same analysis as in (A) for peaks that were more accessible in IL-7Rα/Rag2$^{-/-}$.

IL-7Rα$^{-/-}$ display widespread defects at both stages of *Igh* recombination. Most importantly, the V_H repertoire was highly biased toward 3′ V_H genes. Reduced use of 5′ V_H genes was much more pronounced than in FL pro-B cells, indicating that IL-7R signaling is specifically needed in the BM to make all V_H genes available for the primary antibody repertoire. The presence of N-additions within IL-7Rα$^{-/-}$ VDJ_H sequences demonstrates that they are derived from BM, not FL, progenitors. Our findings concur with a study in neonatal IL-7Rα$^{-/-}$ BM (Hesslein et al., 2006). Detection of N-additions at D_H-J_H junctions indicated that D_H to J_H recombination also took place *de novo* in the BM. Thus, V(D)J recombination progressed with normal dynamics but severely restricted participation of both V_H and D_H genes. Previous models of a block in B cell development in IL-7Rα$^{-/-}$ BM (Kikuchi et al., 2005; Peschon et al., 1994; Miller et al., 2002; Carvalho et al., 2001) inferred that IL-7Rα$^{-/-}$ BM B cells had originated in the FL, where the IL-7R is not essential (Erlandsson et al., 2004), due to their restricted Vh gene usage. Here, our demonstration that V(D)J recombination occurs *de novo* in the BM, albeit in the very few remaining B cells, has enabled us to uncover specific roles of the IL-7R in regulating D_H and V_H gene usage in the *Igh* repertoire in BM pro-B cells.

A previous study showing that IL-7Rα$^{-/-}$ B cell development could be partially rescued by a vav-cre bcl2 transgene, indicating a crucial role in CLP survival (Malin et al., 2010), suggested that IL-7R signaling is not required for BM B cell recombination. However, *Bcl2* driven by the *Igh* Eμ enhancer did not rescue B cell development (Maraskovsky et al., 1998), indicating that the IL-7R has important functions beyond survival, in pro-B cells where V(D)J recombination is taking place. Here, we show that these functions include making the *Igh* locus accessible for V(D)J recombination.

Furthermore, we have uncovered mechanisms underpinning impaired recombination of V_H genes. It must be noted that we performed RNA- and ATAC-seq on a Rag2$^{-/-}$ background. Although an established model for studying mechanisms underpinning recombination because the *Igh* locus remains in an intact, poised state, it has the caveat that D_H to J_H recombination, which normally affects locus structure and V region accessibility, has not occurred. Nevertheless, several marks of V region accessibility are acquired, against which we measured the effect of IL-7R loss. There were no significant differences in local chromatin accessibility over V_H gene RSSs in IL-7Rα/Rag2$^{-/-}$ cells. Importantly, low accessibility at Vκ gene RSSs suggests that surviving IL-7Rα$^{-/-}$ B cells have not "rushed through" to the IL-7R-independent pre-B cell stage where increased Vκ access occurs. IL-7R signaling must be downregulated to enable Igκ recombination (Johnson et al., 2008; Mandal et al., 2011). Here, loss of the IL-7R is not sufficient to activate Vκ genes, suggesting that additional mechanisms are at play (Mandal et al., 2019). Overall, we find no evidence that defects in local accessibility at 5′ V genes account for the preference for recombination of 3′ V_H genes in IL-7Rα$^{-/-}$ cells. We also found that V_H genic non-coding transcription rarely changed in IL-7Rα$^{-/-}$ pro-B cells, supporting multiomics studies that found no correlation with V_H usage (Choi et al., 2013; Bolland et al., 2016).

Non-coding intergenic transcription activates T cell receptor α (TCRα) locus recombination *in vivo* (Abarrategui and Krangel, 2006), although *de novo* antisense transcription over *Igh* 3′ V_H genes increases their recombination (Guo et al., 2011). These and other findings support a model in which intergenic transcription drives recombination (Corcoran, 2010). Here, widespread loss of all PAX5-dependent (PAIRs 4 and 6) and PAX5-independent (J558 and J606) antisense intergenic transcripts (Bolland et al., 2004, 2016; Ebert et al., 2011; Verma-Gaur et al., 2012; Choi et al., 2013) suggests that the IL-7R regulates all *Igh*

CellPress
OPEN ACCESS

antisense transcription and supports a role in promoting long-range mechanisms underpinning V_H to D_H recombination. We did not observe *de novo* antisense transcription over 3′ V_H genes in IL-7Rα$^{-/-}$ pro-B cells, indicating the relative increase in 3′ V_H gene recombination is secondary to the defect in 5′ recombination rather than a bona fide increase in 3′ recombination (Guo et al., 2011).

PAX5, downregulated in IL-7Rα$^{-/-}$ cells, is essential for *Igh* locus contraction (Fuxa et al., 2004; Nutt et al., 1997; Hesslein et al., 2003; Medvedovic et al., 2013; Montefiori et al., 2016). PAX5 has pleiotropic functions, but here, reduced accessibility at Pax5 sites on PAIR promoters and downregulation of PAX5-dependent PAIR transcription in the *Igh* locus suggests that PAX5 binding and function at these regulatory regions is directly impaired by loss of the IL-7R (Ebert et al., 2011).

A key finding was that D_H to J_H recombination is also impaired in IL-7Rα$^{-/-}$ BM B cells. Representation of the central DSP family was severely reduced. Strikingly, antisense non-coding transcription over the D_H region was also ablated. These findings support our model that antisense transcription over the DSP genes activates their recombination (Bolland et al., 2007) and reveal a role for the IL-7R in activating this transcription to drive D_H to J_H recombination.

Reduced accessibility at hundreds of EBF1 binding sites and reduced expression of multiple Pax5 targets by GSEA suggests specific functional consequences of reduced EBF1 and Pax5 expression. Conversely, increased accessibility at putative T cell TF motifs suggests that, although IL-7Rα$^{-/-}$ B cells are committed to the B cell lineage, they nevertheless remain plastic, similarly to PAX5$^{-/-}$ B cells (Nutt et al., 1999).

Our findings have important implications for human B cell development. Pediatric studies suggested early human B cell development did not require IL-7 (LeBien, 2000), but recent studies have shown that adult B cell development is dependent on IL-7R signaling, thereby aligning the dynamics of mouse and human IL-7R dependency (Parrish et al., 2009; Milford et al., 2016). It will be important to determine whether the IL-7R regulates immunoglobulin recombination in human B cells. Human immunodeficiency diseases and aging both have restricted antibody repertoires and poor response to infection (Siegrist and Aspinall, 2009; Martin et al., 2015). Notably, both *Igh* recombination and IL-7R signaling are impaired in aging mice (Stephan et al., 1997; Szabo et al., 1999) and humans. The therapeutic potential of the IL-7R in human aging is an emerging area of interest (Passtoors et al., 2015), and our findings suggest therapeutic potential of IL-7 for boosting naive antibody repertoires.

In conclusion, we reveal that, in addition to its roles in pro-B cell survival and proliferation, IL-7R signaling shapes the *Igh* repertoire at both the D_H-to-J_H and V_H-to-DJ_H stages of recombination in mouse BM and identify several mechanisms by which it can activate the *Igh* locus. IL-7R signaling is therefore essential for expanding antibody diversity to ensure robust activation of the adaptive immune system.

STAR★METHODS

Detailed methods are provided in the online version of this paper and include the following:

- KEY RESOURCES TABLE
- RESOURCE AVAILABILITY
  - Lead contact
  - Materials availability
  - Data and code availability
- EXPERIMENTAL MODEL AND SUBJECT DETAILS
  - Mice
- METHOD DETAILS
  - Primary cells
  - DNA extraction
  - RNA-seq
  - VDJ-seq
  - ATAC-seq
- QUANTIFICATION AND STATISTICAL ANALYSIS

SUPPLEMENTAL INFORMATION

Supplemental information can be found online at https://doi.org/10.1016/j.celrep.2021.109349.

ACKNOWLEDGMENTS

We thank Geoff Butcher, Martin Turner, and members of the Corcoran lab for helpful discussions and critical reading of the manuscript. We thank Mark Veldhoen for provision of IL-7Rα/Rag2$^{-/-}$ mice. We thank Flow Cytometry and Biological Services Unit for excellent support. Work in our laboratory is supported by grants from the BBSRC (BBS/E/B/000C0404, BBS/E/B/000C0405, BBS/E/B/000C0427, BBS/E/B/000C0428, and Core Capability Grant), A.B.-E. was supported by an MRC PhD studentship (1236141), B.A.S. was supported by an MRC PhD studentship (1129229), and M.J.T.S. was supported by a Babraham Institute PhD studentship.

AUTHOR CONTRIBUTIONS

Conceptualization, A.B.-E., B.A.S., and A.E.C.; methodology, A.B.-E., B.A.S., and D.J.B.; software, B.A.S., M.J.T.S., and S.A.; validation, A.B.-E. and A.E.C.; formal analysis, A.B.-E., B.A.S., M.J.T.S., and S.A.; investigation, A.B.-E. and B.A.S.; resources, A.B.-E., K.T., and A.E.C.; data curation, A.B.-E. and B.A.S.; writing – original draft, A.B.-E. and A.E.C.; writing – review and editing, A.B.-E., B.A.S., M.J.T.S., D.J.B., S.A., and A.E.C.; visualization, A.B.-E. and B.A.S.; supervision, A.E.C.; project administration, A.B.-E. and A.E.C.; funding acquisition, A.E.C.

DECLARATION OF INTERESTS

D.J.B. and A.E.C. are inventors on a patent method of identifying VDJ recombination products, published in 2013, PCT/GB2O13/050516 and WO2013128204A1; in 2015, US2015/0031042; and in 2017, US9797014B2. M.J.T.S. is an employee and share- and option-holder of 10x Genomics.

INCLUSION AND DIVERSITY

We worked to ensure sex balance in the selection of non-human subjects. One or more of the authors of this paper self-identifies as an underrepresented ethnic minority in science. While citing references scientifically relevant for this work, we also actively worked to promote gender balance in our reference list.

Received: December 9, 2020
Revised: April 5, 2021
Accepted: June 15, 2021
Published: July 13, 2021

REFERENCES

Abarrategui, I., and Krangel, M.S. (2006). Regulation of T cell receptor-alpha gene recombination by transcription. Nat. Immunol. *7*, 1109–1115.

Bertolino, E., Reddy, K., Medina, K.L., Parganas, E., Ihle, J., and Singh, H. (2005). Regulation of interleukin 7-dependent immunoglobulin heavy-chain variable gene rearrangements by transcription factor STAT5. Nat. Immunol. *6*, 836–843.

Bolland, D.J., Wood, A.L., Johnston, C.M., Bunting, S.F., Morgan, G., Chakalova, L., Fraser, P.J., and Corcoran, A.E. (2004). Antisense intergenic transcription in V(D)J recombination. Nat. Immunol. *5*, 630–637.

Bolland, D.J., Wood, A.L., Afshar, R., Featherstone, K., Oltz, E.M., and Corcoran, A.E. (2007). Antisense intergenic transcription precedes Igh D-to-J recombination and is controlled by the intronic enhancer Emu. Mol. Cell. Biol. *27*, 5523–5533.

Bolland, D.J., Koohy, H., Wood, A.L., Matheson, L.S., Krueger, F., Stubbington, M.J.T., Baizan-Edge, A., Chovanec, P., Stubbs, B.A., Tabbada, K., et al. (2016). Two mutually exclusive local chromatin states drive efficient V(D)J recombination. Cell Rep. *15*, 2475–2487.

Boller, S., and Grosschedl, R. (2014). The regulatory network of B-cell differentiation: a focused view of early B-cell factor 1 function. Immunol. Rev. *261*, 102–115.

Buenrostro, J.D., Giresi, P.G., Zaba, L.C., Chang, H.Y., and Greenleaf, W.J. (2013). Transposition of native chromatin for fast and sensitive epigenomic profiling of open chromatin, DNA-binding proteins and nucleosome position. Nat. Methods *10*, 1213–1218.

Buenrostro, J.D., Wu, B., Chang, H.Y., and Greenleaf, W.J. (2015). ATAC-seq: a method for assaying chromatin accessibility genome-wide. Curr. Protoc. Mol. Biol. *109*, 21.29.1–21.29.9.

Carvalho, T.L., Mota-Santos, T., Cumano, A., Demengeot, J., and Vieira, P. (2001). Arrested B lymphopoiesis and persistence of activated B cells in adult interleukin 7(-/)- mice. J. Exp. Med. *194*, 1141–1150.

Choi, N.M., Loguercio, S., Verma-Gaur, J., Degner, S.C., Torkamani, A., Su, A.I., Oltz, E.M., Artyomov, M., and Feeney, A.J. (2013). Deep sequencing of the murine IgH repertoire reveals complex regulation of nonrandom V gene rearrangement frequencies. J. Immunol. *191*, 2393–2402.

Chovanec, P., Bolland, D.J., Matheson, L.S., Wood, A.L., Krueger, F., Andrews, S., and Corcoran, A.E. (2018). Unbiased quantification of immunoglobulin diversity at the DNA level with VDJ-seq. Nat. Protoc. *13*, 1232–1252.

Chowdhury, D., and Sen, R. (2001). Stepwise activation of the immunoglobulin mu heavy chain gene locus. EMBO J. *20*, 6394–6403.

Chowdhury, D., and Sen, R. (2003). Transient IL-7/IL-7R signaling provides a mechanism for feedback inhibition of immunoglobulin heavy chain gene rearrangements. Immunity *18*, 229–241.

Corcoran, A.E. (2010). The epigenetic role of non-coding RNA transcription and nuclear organization in immunoglobulin repertoire generation. Semin. Immunol. *22*, 353–361.

Corcoran, A.E., Smart, F.M., Cowling, R.J., Crompton, T., Owen, M.J., and Venkitaraman, A.R. (1996). The interleukin-7 receptor alpha chain transmits distinct signals for proliferation and differentiation during B lymphopoiesis. EMBO J. *15*, 1924–1932.

Corcoran, A.E., Riddell, A., Krooshoop, D., and Venkitaraman, A.R. (1998). Impaired immunoglobulin gene rearrangement in mice lacking the IL-7 receptor. Nature *391*, 904–907.

Corfe, S.A., and Paige, C.J. (2012). The many roles of IL-7 in B cell development; mediator of survival, proliferation and differentiation. Semin. Immunol. *24*, 198–208.

Decker, T., Pasca di Magliano, M., McManus, S., Sun, Q., Bonifer, C., Tagoh, H., and Busslinger, M. (2009). Stepwise activation of enhancer and promoter regions of the B cell commitment gene Pax5 in early lymphopoiesis. Immunity *30*, 508–520.

Dias, S., Silva, H., Jr., Cumano, A., and Vieira, P. (2005). Interleukin-7 is necessary to maintain the B cell potential in common lymphoid progenitors. J. Exp. Med. *201*, 971–979.

Ebert, A., McManus, S., Tagoh, H., Medvedovic, J., Salvagiotto, G., Novatchkova, M., Tamir, I., Sommer, A., Jaritz, M., and Busslinger, M. (2011). The distal V(H) gene cluster of the Igh locus contains distinct regulatory elements with Pax5 transcription factor-dependent activity in pro-B cells. Immunity *34*, 175–187.

Erlandsson, L., Licence, S., Gaspal, F., Bell, S., Lane, P., Corcoran, A.E., and Mårtensson, I.L. (2004). Impaired B-1 and B-2 B cell development and atypical splenic B cell structures in IL-7 receptor-deficient mice. Eur. J. Immunol. *34*, 3595–3603.

Feeney, A.J. (1990). Lack of N regions in fetal and neonatal mouse immunoglobulin V-D-J junctional sequences. J. Exp. Med. *172*, 1377–1390.

Fuxa, M., Skok, J., Souabni, A., Salvagiotto, G., Roldan, E., and Busslinger, M. (2004). Pax5 induces V-to-DJ rearrangements and locus contraction of the immunoglobulin heavy-chain gene. Genes Dev. *18*, 411–422.

Gerasimova, T., Guo, C., Ghosh, A., Qiu, X., Montefiori, L., Verma-Gaur, J., Choi, N.M., Feeney, A.J., and Sen, R. (2015). A structural hierarchy mediated by multiple nuclear factors establishes IgH locus conformation. Genes Dev. *29*, 1683–1695.

Guo, C., Yoon, H.S., Franklin, A., Jain, S., Ebert, A., Cheng, H.-L., Hansen, E., Despo, O., Bossen, C., Vettermann, C., et al. (2011). CTCF-binding elements mediate control of V(D)J recombination. Nature *477*, 424–430.

Györy, I., Boller, S., Nechanitzky, R., Mandel, E., Pott, S., Liu, E., and Grosschedl, R. (2012). Transcription factor Ebf1 regulates differentiation stage-specific signaling, proliferation, and survival of B cells. Genes Dev. *26*, 668–682.

Heinz, S., Benner, C., Spann, N., Bertolino, E., Lin, Y.C., Laslo, P., Cheng, J.X., Murre, C., Singh, H., and Glass, C.K. (2010). Simple combinations of lineage-determining transcription factors prime cis-regulatory elements required for macrophage and B cell identities. Mol. Cell *38*, 576–589.

Hesslein, D.G.T., Pflugh, D.L., Chowdhury, D., Bothwell, A.L.M., Sen, R., and Schatz, D.G. (2003). Pax5 is required for recombination of transcribed, acetylated, 5′ IgH V gene segments. Genes Dev. *17*, 37–42.

Hesslein, D.G.T., Yang, S.Y., and Schatz, D.G. (2006). Origins of peripheral B cells in IL-7 receptor-deficient mice. Mol. Immunol. *43*, 326–334.

Jain, S., Ba, Z., Zhang, Y., Dai, H.-Q., and Alt, F.W. (2018). CTCF-binding elements mediate accessibility of Rag substrates during chromatin scanning. Cell *174*, 102–116.e14.

Jeong, H.D., and Teale, J.M. (1988). Comparison of the fetal and adult functional B cell repertoires by analysis of VH gene family expression. J. Exp. Med. *168*, 589–603.

Jhunjhunwala, S., van Zelm, M.C., Peak, M.M., Cutchin, S., Riblet, R., van Dongen, J.J.M., Grosveld, F.G., Knoch, T.A., and Murre, C. (2008). The 3D structure of the immunoglobulin heavy-chain locus: implications for long-range genomic interactions. Cell *133*, 265–279.

Ji, Y., Resch, W., Corbett, E., Yamane, A., Casellas, R., and Schatz, D.G. (2010). The in vivo pattern of binding of RAG1 and RAG2 to antigen receptor loci. Cell *141*, 419–431.

Johnson, K., Angelin-Duclos, C., Park, S., and Calame, K.L. (2003). Changes in histone acetylation are associated with differences in accessibility of V(H) gene segments to V-DJ recombination during B-cell ontogeny and development. Mol. Cell. Biol. *23*, 2438–2450.

Johnson, K., Hashimshony, T., Sawai, C.M., Pongubala, J.M.R., Skok, J.A., Aifantis, I., and Singh, H. (2008). Regulation of immunoglobulin light-chain recombination by the transcription factor IRF-4 and the attenuation of interleukin-7 signaling. Immunity *28*, 335–345.

Johnson, K., Chaumeil, J., Micsinai, M., Wang, J.M.H., Ramsey, L.B., Baracho, G.V., Rickert, R.C., Strino, F., Kluger, Y., Farrar, M.A., et al. (2012). IL-7 functionally segregates the pro-B cell stage by regulating transcription of recombination mediators across cell cycle. J. Immunol. *188*, 6084–6092.

Johnston, C.M., Wood, A.L., Bolland, D.J., and Corcoran, A.E. (2006). Complete sequence assembly and characterization of the C57BL/6 mouse Ig heavy chain V region. J. Immunol. *176*, 4221–4234.

Kikuchi, K., Lai, A.Y., Hsu, C.-L., and Kondo, M. (2005). IL-7 receptor signaling is necessary for stage transition in adult B cell development through up-regulation of EBF. J. Exp. Med. *201*, 1197–1203.

Langmead, B., and Salzberg, S.L. (2012). Fast gapped-read alignment with Bowtie 2. Nat. Methods *9*, 357–359.

LeBien, T.W. (2000). Fates of human B-cell precursors. Blood *96*, 9–23.

Lefranc, M.-P., Giudicelli, V., Duroux, P., Jabado-Michaloud, J., Folch, G., Aouinti, S., Carillon, E., Duvergey, H., Houles, A., Paysan-Lafosse, T., et al. (2015). IMGT®, the international ImMunoGeneTics information system® 25 years on. Nucleic Acids Res. *43* (Database issue, D1), D413–D422.

Li, Y.S., Hayakawa, K., and Hardy, R.R. (1993). The regulated expression of B lineage associated genes during B cell differentiation in bone marrow and fetal liver. J. Exp. Med. *178*, 951–960.

Lin, Y.C., Jhunjhunwala, S., Benner, C., Heinz, S., Welinder, E., Mansson, R., Sigvardsson, M., Hagman, J., Espinoza, C.A., Dutkowski, J., et al. (2010). A global network of transcription factors, involving E2A, EBF1 and Foxo1, that orchestrates B cell fate. Nat. Immunol. *11*, 635–643.

Love, M.I., Huber, W., and Anders, S. (2014). Moderated estimation of fold change and dispersion for RNA-seq data with DESeq2. Genome Biol. *15*, 550.

Malin, S., McManus, S., Cobaleda, C., Novatchkova, M., Delogu, A., Bouillet, P., Strasser, A., and Busslinger, M. (2010). Role of STAT5 in controlling cell survival and immunoglobulin gene recombination during pro-B cell development. Nat. Immunol. *11*, 171–179.

Mandal, M., Powers, S.E., Maienschein-Cline, M., Bartom, E.T., Hamel, K.M., Kee, B.L., Dinner, A.R., and Clark, M.R. (2011). Epigenetic repression of the Igk locus by STAT5-mediated recruitment of the histone methyltransferase Ezh2. Nat. Immunol. *12*, 1212–1220.

Mandal, M., Okoreeh, M.K., Kennedy, D.E., Maienschein-Cline, M., Ai, J., McLean, K.C., Kaverina, N., Veselits, M., Aifantis, I., Gounari, F., and Clark, M.R. (2019). CXCR4 signaling directs Igk recombination and the molecular mechanisms of late B lymphopoiesis. Nat. Immunol. *20*, 1393–1403.

Maraskovsky, E., Peschon, J.J., McKenna, H., Teepe, M., and Strasser, A. (1998). Overexpression of Bcl-2 does not rescue impaired B lymphopoiesis in IL-7 receptor-deficient mice but can enhance survival of mature B cells. Int. Immunol. *10*, 1367–1375.

Martin, V., Bryan Wu, Y.C., Kipling, D., and Dunn-Walters, D. (2015). Ageing of the B-cell repertoire. Philos. Trans. R. Soc. Lond. B Biol. Sci. *370*, 20140237.

Matheson, L.S., Bolland, D.J., Chovanec, P., Krueger, F., Andrews, S., Koohy, H., and Corcoran, A.E. (2017). Local chromatin features including PU.1 and IKAROS binding and H3K4 methylation shape the repertoire of immunoglobulin kappa genes chosen for V(D)J recombination. Front. Immunol. *8*, 1550.

Medina, K.L., Tangen, S.N., Seaburg, L.M., Thapa, P., Gwin, K.A., and Shapiro, V.S. (2013). Separation of plasmacytoid dendritic cells from B-cell-biased lymphoid progenitor (BLP) and Pre-pro B cells using PDCA-1. PLoS ONE *8*, e78408.

Medvedovic, J., Ebert, A., Tagoh, H., Tamir, I.M., Schwickert, T.A., Novatchkova, M., Sun, Q., Huis In 't Veld, P.J., Guo, C., Yoon, H.S., et al. (2013). Flexible long-range loops in the VH gene region of the Igh locus facilitate the generation of a diverse antibody repertoire. Immunity *39*, 229–244.

Milford, T.A., Su, R.J., Francis, O.L., Baez, I., Martinez, S.R., Coats, J.S., Weldon, A.J., Calderon, M.N., Nwosu, M.C., Botimer, A.R., et al. (2016). TSLP or IL-7 provide an IL-7Rα signal that is critical for human B lymphopoiesis. Eur. J. Immunol. *46*, 2155–2161.

Miller, J.P., Izon, D., DeMuth, W., Gerstein, R., Bhandoola, A., and Allman, D. (2002). The earliest step in B lineage differentiation from common lymphoid progenitors is critically dependent upon interleukin 7. J. Exp. Med. *196*, 705–711.

Montefiori, L., Wuerffel, R., Roqueiro, D., Lajoie, B., Guo, C., Gerasimova, T., De, S., Wood, W., Becker, K.G., Dekker, J., et al. (2016). Extremely long-range chromatin loops link topological domains to facilitate a diverse antibody repertoire. Cell Rep. *14*, 896–906.

Nutt, S.L., Urbánek, P., Rolink, A., and Busslinger, M. (1997). Essential functions of Pax5 (BSAP) in pro-B cell development: difference between fetal and adult B lymphopoiesis and reduced V-to-DJ recombination at the IgH locus. Genes Dev. *11*, 476–491.

Nutt, S.L., Heavey, B., Rolink, A.G., and Busslinger, M. (1999). Commitment to the B-lymphoid lineage depends on the transcription factor Pax5. Nature *401*, 556–562.

O'Riordan, M., and Grosschedl, R. (1999). Coordinate regulation of B cell differentiation by the transcription factors EBF and E2A. Immunity *11*, 21–31.

Parkhomchuk, D., Borodina, T., Amstislavskiy, V., Banaru, M., Hallen, L., Krobitsch, S., Lehrach, H., and Soldatov, A. (2009). Transcriptome analysis by strand-specific sequencing of complementary DNA. Nucleic Acids Res. *37*, e123.

Parrish, Y.K., Baez, I., Milford, T.-A., Benitez, A., Galloway, N., Rogerio, J.W., Sahakian, E., Kagoda, M., Huang, G., Hao, Q.-L., et al. (2009). IL-7 dependence in human B lymphopoiesis increases during progression of ontogeny from cord blood to bone marrow. J. Immunol. *182*, 4255–4266.

Passtoors, W.M., van den Akker, E.B., Deelen, J., Maier, A.B., van der Breggen, R., Jansen, R., Trompet, S., van Heemst, D., Derhovanessian, E., Pawelec, G., et al. (2015). IL7R gene expression network associates with human healthy ageing. Immun. Ageing *12*, 21.

Peschon, J.J., Morrissey, P.J., Grabstein, K.H., Ramsdell, F.J., Maraskovsky, E., Gliniak, B.C., Park, L.S., Ziegler, S.F., Williams, D.E., Ware, C.B., et al. (1994). Early lymphocyte expansion is severely impaired in interleukin 7 receptor-deficient mice. J. Exp. Med. *180*, 1955–1960.

Pongubala, J.M.R., Northrup, D.L., Lancki, D.W., Medina, K.L., Treiber, T., Bertolino, E., Thomas, M., Grosschedl, R., Allman, D., and Singh, H. (2008). Transcription factor EBF restricts alternative lineage options and promotes B cell fate commitment independently of Pax5. Nat. Immunol. *9*, 203–215.

Pridans, C., Holmes, M.L., Polli, M., Wettenhall, J.M., Dakic, A., Corcoran, L.M., Smyth, G.K., and Nutt, S.L. (2008). Identification of Pax5 target genes in early B cell differentiation. J. Immunol. *180*, 1719–1728.

Pulivarthy, S.R., Lion, M., Kuzu, G., Matthews, A.G., Borowsky, M.L., Morris, J., Kingston, R.E., Dennis, J.H., Tolstorukov, M.Y., and Oettinger, M.A. (2016). Regulated large-scale nucleosome density patterns and precise nucleosome positioning correlate with V(D)J recombination. Proc. Natl. Acad. Sci. USA *113*, E6427–E6436.

Revilla-I-Domingo, R., Bilic, I., Vilagos, B., Tagoh, H., Ebert, A., Tamir, I.M., Smeenk, L., Trupke, J., Sommer, A., Jaritz, M., and Busslinger, M. (2012). The B-cell identity factor Pax5 regulates distinct transcriptional programmes in early and late B lymphopoiesis. EMBO J. *31*, 3130–3146.

Rochman, Y., Spolski, R., and Leonard, W.J. (2009). New insights into the regulation of T cells by γ(c) family cytokines. Nat. Rev. Immunol. *9*, 480–490.

Roessler, S., Györy, I., Imhof, S., Spivakov, M., Williams, R.R., Busslinger, M., Fisher, A.G., and Grosschedl, R. (2007). Distinct promoters mediate the regulation of Ebf1 gene expression by interleukin-7 and Pax5. Mol. Cell. Biol. *27*, 579–594.

Rumfelt, L.L., Zhou, Y., Rowley, B.M., Shinton, S.A., and Hardy, R.R. (2006). Lineage specification and plasticity in CD19- early B cell precursors. J. Exp. Med. *203*, 675–687.

Sayegh, C.E., Jhunjhunwala, S., Riblet, R., and Murre, C. (2005). Visualization of looping involving the immunoglobulin heavy-chain locus in developing B cells. Genes Dev. *19*, 322–327.

Shinkai, Y., Rathbun, G., Lam, K.P., Oltz, E.M., Stewart, V., Mendelsohn, M., Charron, J., Datta, M., Young, F., Stall, A.M., et al. (1992). RAG-2-deficient mice lack mature lymphocytes owing to inability to initiate V(D)J rearrangement. Cell *68*, 855–867.

Siegrist, C.-A., and Aspinall, R. (2009). B-cell responses to vaccination at the extremes of age. Nat. Rev. Immunol. *9*, 185–194.

Stephan, R.P., Lill-Elghanian, D.A., and Witte, P.L. (1997). Development of B cells in aged mice: decline in the ability of pro-B cells to respond to IL-7 but not to other growth factors. J. Immunol. *158*, 1598–1609.

Stubbington, M.J., and Corcoran, A.E. (2013). Non-coding transcription and large-scale nuclear organisation of immunoglobulin recombination. Curr. Opin. Genet. Dev. *23*, 81–88.

Subramanian, A., Tamayo, P., Mootha, V.K., Mukherjee, S., Ebert, B.L., Gillette, M.A., Paulovich, A., Pomeroy, S.L., Golub, T.R., Lander, E.S., and Mesirov, J.P. (2005). Gene set enrichment analysis: a knowledge-based approach for interpreting genome-wide expression profiles. Proc. Natl. Acad. Sci. USA *102*, 15545–15550.

Szabo, P., Shen, S., and Weksler, M.E. (1999). Age-associated defects in B lymphocyte development. Exp. Gerontol. *34*, 431–434.

Tsapogas, P., Zandi, S., Åhsberg, J., Zetterblad, J., Welinder, E., Jönsson, J.I., Månsson, R., Qian, H., and Sigvardsson, M. (2011). IL-7 mediates Ebf-1-dependent lineage restriction in early lymphoid progenitors. Blood *118*, 1283–1290.

Verma-Gaur, J., Torkamani, A., Schaffer, L., Head, S.R., Schork, N.J., and Feeney, A.J. (2012). Noncoding transcription within the Igh distal V(H) region at PAIR elements affects the 3D structure of the Igh locus in pro-B cells. Proc. Natl. Acad. Sci. USA *109*, 17004–17009.

Vilagos, B., Hoffmann, M., Souabni, A., Sun, Q., Werner, B., Medvedovic, J., Bilic, I., Minnich, M., Axelsson, E., Jaritz, M., and Busslinger, M. (2012). Essential role of EBF1 in the generation and function of distinct mature B cell types. J. Exp. Med. *209*, 775–792.

Vosshenrich, C.A., Cumano, A., Müller, W., Di Santo, J.P., and Vieira, P. (2003). Thymic stromal-derived lymphopoietin distinguishes fetal from adult B cell development. Nat. Immunol. *4*, 773–779.

Xu, C.-R., Schaffer, L., Head, S.R., and Feeney, A.J. (2008). Reciprocal patterns of methylation of H3K36 and H3K27 on proximal vs. distal IgVH genes are modulated by IL-7 and Pax5. Proc. Natl. Acad. Sci. USA *105*, 8685–8690.

Yancopoulos, G.D., and Alt, F.W. (1985). Developmentally controlled and tissue-specific expression of unrearranged VH gene segments. Cell *40*, 271–281.

Yancopoulos, G.D., Malynn, B.A., and Alt, F.W. (1988). Developmentally regulated and strain-specific expression of murine VH gene families. J. Exp. Med. *168*, 417–435.

Yu, M., Chen, Y., Zeng, H., Zheng, Y., Fu, G., Zhu, W., Broeckel, U., Aggarwal, P., Turner, A., Neale, G., et al. (2017). PLCγ-dependent mTOR signalling controls IL-7-mediated early B cell development. Nat. Commun. *8*, 1457.

Zhang, Y., Liu, T., Meyer, C.A., Eeckhoute, J., Johnson, D.S., Bernstein, B.E., Nusbaum, C., Myers, R.M., Brown, M., Li, W., and Liu, X.S. (2008). Model-based analysis of ChIP-Seq (MACS). Genome Biol. *9*, R137.

CellPress
OPEN ACCESS

STAR★METHODS

KEY RESOURCES TABLE

REAGENT or RESOURCE	SOURCE	IDENTIFIER
Antibodies		
CD11b Monoclonal Antibody, Biotin	eBioscience	Clone M1/70; Cat# 13-0112-82; RRID:AB_466359
Ly-6G/Gr-1 Monoclonal Antibody, Biotin	eBioscience	Clone RB6-8C5; Cat# 13-5931-82; RRID: AB_466800
RAT ANTI MOUSE Ly-6C:Biotin	AbD Serotec	Clone ER-MP20 Cat# MCA2389B RRID: AB_844550
TER-119 Monoclonal Antibody, Biotin	eBioscience	Clone TER-119 Cat# 13-5921-82 RRID: AB_466797
CD3e Monoclonal Antibody, Biotin	eBioscience	Clone 145-2C11 Cat# 13-0031-82 RRID: AB_466319
BV421 Rat Anti-Mouse CD45R/B220	BD bioscience	Clone RA3-6B2 Cat# 562922 RRID: AB_2737894
PerCP-Cy5.5 Rat Anti-Mouse CD19	BD bioscience	Clone 1D3 Cat# 561113 RRID: AB_10563071
FITC Rat Anti-Mouse CD43	BD bioscience	Clone S7 Cat# 553270 RRID: AB_394747
Chemicals, peptides and recombinant proteins		
Agencourt AMPure XP beads	Beckman	Cat# A63880
Critical commercial assays		
Ovation RNA-seq SystemV2 kit	NuGen	Cat# 7102-08
TruSeq RNA Library Prep Kit v2	Illumina	Cat# RS-122-2001
Nextera DNA Sample Preparation Kit	Illumina	Cat# 15028211
Deposited data		
Raw and analyzed data	This paper	GEO: GSE157603
PAX5 ChIP Rag$^{-/-}$ pro-B	Revilla-I-Domingo et al., 2012	GEO: GSM932924
EBF1 ChIP Rag$^{-/-}$ pro-B	Vilagos et al., 2012	GEO: GSM876622, GSM876623
Experimental models: organisms/strains		
Mouse: IL7R$\alpha^{-/-}$	J.J. Peschon	Peschon et al., 1994
Mouse: RAG2$^{-/-}$	Frederick Alt	Shinkai et al., 1992
Software and algorithms		
Bowtie2	Langmead and Salzberg, 2012	http://bowtie-bio.sourceforge.net/bowtie2/index.shtml
Seqmonk	The Babraham Institute	https://www.bioinformatics.babraham.ac.uk/projects/seqmonk/
DESeq2	Love et al., 2014	https://bioconductor.org/packages/release/bioc/html/DESeq2.html
LinkON	The Babraham Institute Chovanec et al., 2018	https://github.com/peterch405/BabrahamLinkON/blob/master/README.md
HOMER	Heinz et al., 2010	http://homer.ucsd.edu/homer/ngs/peaks.html
IMGT	Lefranc et al., 2015	http://www.imgt.org/
GSEA4.1	Subramanian et al., 2005	http://www.gsea-msigdb.org/gsea/index.jsp

RESOURCE AVAILABILITY

Lead contact

Further information and requests for resources and reagents should be directed to and will be fulfilled by the Lead Contact, Anne Corcoran (anne.corcoran@babraham.ac.uk).

Materials availability

This study did not generate new unique reagents.

Data and code availability

- The VDJ-seq, ATAC-seq and RNA-seq raw sequencing files generated in this study, as well as processed files have been deposited at GEO, and are publicly available as of the date of publication. Accession numbers are listed in the Key resources table. This paper analyzes existing, publicly available data. These accession numbers for the datasets are listed in the Key resources table.
- This paper does not report original code.
- Any additional information required to reanalyze the data reported in this paper is available from the lead contact upon request.

EXPERIMENTAL MODEL AND SUBJECT DETAILS

Mice

Wild-type, RAG2$^{-/-}$ (Shinkai et al., 1992), IL-7R$\alpha^{-/-}$ (Peschon et al., 1994) and IL-7R$\alpha^{-/-}$ crossed with RAG2$^{-/-}$ (IL-7R$\alpha^{-/-}$ x RAG2$^{-/-}$) C57BL/6 mice were maintained in accordance with Babraham Institute Animal Welfare and Ethical Review Body and Home Office rules under Project License 80/2529. Recommended ARRIVE reporting guidelines were followed. Mice were bred and maintained in the Babraham Institute Biological Services Unit under Specific Opportunistic Pathogen Free (SOPF) conditions. After weaning, mice were maintained in individually ventilated cages (2–5 mice per cage). Mice were fed CRM (P) VP diet (Special Diet Services) *ad libitum*, and millet, sunflower or poppy seeds at cage-cleaning as environmental enrichment. Health status was monitored closely and any mouse with clinical signs of ill-health or distress persisting for more than three days was culled. Treatment with antibiotics was not permitted to avoid interference with immune function. Thus, all mice remained 'sub-threshold' under UK Home Office severity categorization. 6-8-week-old IL-7R$\alpha^{-/-}$ and IL-7R$\alpha^{-/-}$ x RAG2$^{-/-}$ mice (all mixed sex), and 10-12 week old RAG2$^{-/-}$ mice (one female replicate and one male replicate) were used. Although wild-type (WT) comparison data were from 12 week old mice, IL-7R$\alpha^{-/-}$ animals were taken before 10 weeks because they produce fewer BM B cells as they age, with very few produced after 10 weeks (Peschon et al., 1994; Erlandsson et al., 2004). To maximize cell numbers and considering IL-7R$\alpha^{-/-}$ mice as young as 3 weeks have adult B cell populations (Hesslein et al., 2006), pro-B cells from 6-8-week old mice were taken for sorting. Fetal livers (FL) were harvested from day 15.5 mouse embryos.

METHOD DETAILS

Primary cells

Following CO_2 asphyxiation and cervical dislocation, mouse BM was flushed from femurs and tibias, resuspended at 25 × 10^6 cells/ml, and depleted of macrophages, granulocytes, erythroid lineage and T cells using biotinylated antibodies against CD11b (MAC-1; ebioscience; 1:1600), Ly6G (Gr-1; ebioscience; 1:1600), Ly6C (Abd Serotec; 1:400), Ter119 (ebioscience; 1:400) and CD3e (ebioscience; 1:800), incubated on ice for 30 mins, followed by streptavidin MACs beads (10 μl/10^7 cells in 100 μl) (Miltenyi) at 4°C for 15 mins. MACS LS columns were equilibrated and the flow through collected for flow sorting. MACS depletion for FL was carried out as for BM, using TER119-biotin at a higher concentration (1:200). Cells were stained for 45 mins on ice in the dark with the following sorting antibodies from BD Bioscience: BV421-anti B220,1:200; PerCP-Cy5.5-antiCD19, 1:400; FITC-anti CD43, 1:200) Thereafter, pro-B cells from IL-7R$\alpha^{-/-}$ BM and WT FL were flow-sorted for forward and side scatter and cell surface markers as B220$^+$ CD19$^+$, while IL-7R$\alpha^{-/-}$ x RAG2$^{-/-}$ and RAG2$^{-/-}$ BM B pro-B cells were sorted as a B220$^+$ CD19$^+$ CD43$^+$ population on a BD FACSAria in the Babraham Institute Flow Cytometry facility.

DNA extraction

Genomic DNA was isolated from mouse B cells using the DNeasy kit (QIAGEN) according to the manufacturer's instructions, except for the incubation step, which was changed to 30 mins. DNA was eluted in nuclease-free water and quantified by Nanodrop.

RNA-seq

Total RNA was extracted from ~200,000 cells for each replicate using the RNeasy Plus kit (QIAGEN). cDNA preparation was performed using the Ovation RNaseq System V2 kit (NuGen) protocol, and 200 ng of cDNA (made up to 130 μL with nuclease-free water) was carried through to generate 50bp paired-end RNA-seq libraries for Illumina sequencing. cDNA was sonicated using a Covaris E220 to fragment lengths between 200-700 bp (10% duty cycle, 140W peak incident power, 200 cycles per burst, 80 s processing time). End repair was carried out by adding 16 μL of 10x T4 Ligase Buffer (NEB), 4 μL of 10 mM dNTP mix, 3 μL T4 DNA polymerase (5 U/μl, Invitrogen), 1 μL Klenow (2U/μl, Invitrogen), 1 μL T4 polynucleotide kinase (10 U/μl, NEB) and 3 μL nuclease-free water, and incubating the reaction for 30 min at 20°C. Samples were purified again using QIAquick columns and eluted in 43 μL of nuclease-free water. A-tailing was then performed by adding 5 μL of 10x Klenow Buffer (NEB), 1 μL of dATP (10 mM) and 1 μL of exo minus Klenow (5U/μl, Fermentas); the reaction was incubated at 37°C for 30 min and purified using MinElute PCR purification columns (QIAGEN) and cDNA eluted in 10 μl. To sequence multiple libraries in one lane, Illumina TruSeq adaptors (6 bp index) (TruSeq RNA Library Prep Kit v2) were ligated by adding 15 μL 2x Rapid Ligation Buffer (Enzymatics), 4 μL of Rapid T4 DNA Ligase (6U/μl, Enzymatics) and 1 μL of adaptor mix (indexed adaptor and universal adaptor at 1.5 uM each) to the 10 μL of eluted cDNA. This reaction was incubated at

23°C for 30 min and 15°C for 30 min. Agencourt AMPure XP Beads (Beckman Coulter) were used to select library fragments between 200-700 bp by performing double-sided SPRI bead selection. Amplification was performed as follows: a 50 μL reaction was set up by adding 5ul of 10x Pfx amplification buffer (Invitrogen), 0.8 μL Pfx Platinum (2.5 U/μl), 2 μL of dNTPs (10 mM each), 2 μL MgSO4 (50 mM) and 1 μL of each Illumina paired-end primer (25 uM, Sigma) to the 38.2 μL library. Program: 94°C for 2 min, 8-11 cycles of 94°C for 15 s, 62°C for 30 s and 72°C for 30 s, and a final 10 min at 72°C. The library was purified by single-sided SPRI selection as above (modified from Parkhomchuk et al., [2009]). Libraries were sequenced on an Illumina HiSeq2500 (4 libraries per lane).

VDJ-seq

VDJ-seq was carried out as previously described in Bolland et al. (2016). 1-5 μg of DNA was sonicated using a Covaris E220, to get an average length of 500 bp. End repair was carried out by adding 16 μL 10x T4 DNA ligas buffer (NRB), 4 μL dNTP mix (10 mM total each - dATP, dCTP, dGTP and dTTP), 5 μL T4 DNA polymerase (3U/μl, NEB), 1 μL Klenow (5 U/μl, NEB) and 5 μL T4 PNK (10 U/μl, NEB) to the sonicated DNA (161 μL reaction), and incubating it at room temperature for 30 min. The sample was purified following the QIAquick PCR purification column protocol (QIAGEN). Repaired DNA fragments were eluted in 50 μL of nuclease-free water, and A-tailing was then carried out by adding 6 μL of 10x buffer 2 (NEB), 1 μL dATP (10 nM) and 3 μL Klenow exo- (5U/μl, NEB), and incubating the mix at 37°C for 30 min. DNA was purified using QIAquick PCR columns again. Two altered PE1 adaptor mixes were used, both including 6 random nucleotide barcodes. For the latter reaction, the A-tailed samples were split in two, each ligating to one of the two adaptor mixes. Adaptor ligation was carried out by adding 6 μL 10x T4 DNA ligase buffer (NEB), 4 μL adaptor (50 μM) and 5 μL T4 DNA ligase (400,000 U/ml, NEB) to each sample, making a 60 μL reaction. Reactions were incubated at 16°C overnight. The split reactions were pooled after incubation, and QIAquick columns were again used for purification. Depletion of unrecombined sequences was achieved by the use of 4 pairs of biotinylated primers (Bolland et al., 2016) which target the intergenic region upstream of each J_H gene. Each sample was split so that each aliquot was < 1 μg of DNA in 50 μL containing 5 μL 10x ThermoPol reaction buffer (Roche), 2 μL dNTP (10 mM each) and 1 μL Vent exo- (200U/ml, NEB). Incubation: 95°C- 4 min, 55°C- 5 min, 72°C- 15 min. Reactions for each sample were pooled and purified using QIAquick columns. Unrecobined J_H sequences were removed from the samples using streptavidin magnetic beads (Dynabeads MyOne Streptavidin C1, Invitrogen) and samples were purified using QIAquick columns. To enrich for recombined sequences, primer extension was carried out as above (annealing temperature 59°C rather than 55°C) using biotinylated reverse primers for each of the four J_H genes. Recombined sequences were recovered using streptavidin beads as above. Beads were washed twice using 100 μL of washing buffer and once with EB buffer, and resuspended in 42 μL of EB buffer. To amplify the library, a PE1 primer (Illumina) and a mix of four J_H primers which included a PE2 adaptor sequence (Bolland et at., 2016), were used. The resuspended beads were split into four aliquots (10.5 μL each) and 12.5 μL Pwo master mix (Sigma-Aldrich), 1 μL PE1 primer (10 μM) and 1 μL JH reverse primer mix (10 μM) were added to make a 25 μL reaction. Incubation: 94°C- 2 min, 15 cycles of 94°C- 15 s, 61°C-30 s, 72°C- 45 s, followed by a final 72°C- 5 min. The sample was pooled again, and beads were washed using 30 μL EB (QIAGEN), which was added to the supernatant containing the amplified library. These were put through a double-sided selection using Ampure XP beads (Bekman Coulter) for 200-700 bp. The library was further amplified to incorporate flowcell-binding indexes. PE1 and PE2 (including the index) primers (Illumina) were added to a PCR reaction as described above but with only 5 cycles and an annealing temperature of 55°C, rather than 61°C. PCR products were combined and purified using SPRI beads, eluting in 20 μl.

Libraries were sequenced on an Illumina HiSeq (8-12 libraries/lane; 250 BP paired-end). Due to the low number of cells in IL-7R$\alpha^{-/-}$ BM, VDJ-seq libraries were generated with approximately 6-fold less starting material, resulting in reduced numbers of sequences relative to the WT BM VDJ-seq libraries (Table S7). Nevertheless, this amount of starting material does not compromise detection of the wide dynamic range of frequency of VDJ and DJ recombined sequences (Chovanec et al., 2018).

ATAC-seq

ATAC-seq (Assay for Transposase-Accessible Chromatin using sequencing) was performed as previously described (Buenrostro et al., 2013, 2015) on 70,000-100,000 cells. Sorted cells were washed, centrifuged, and resuspended in 50 μl of lysis buffer (10 mM Tris-HCl pH 7.4, 10 mM NaCl, 3 mM MgCl2, 0.1% NP40) on ice for 15 min. Nuclei were centrifuged at 600 r*cf.* for 10 min at 4°C, resuspended in 50 μl 1xTD buffer containing 2.5 μl TDE1 transposase (Illumina Nextera DNA Sample Preparation Kit), and incubated for 30 min at 37°C. Samples were purified using MiniElute columns (QIAGEN) according to the manufacturer's instructions and eluted in 21 μl RSB buffer (10mM Tris HCl pH7.6, 10mM NaCl, 1.5mM MgCl2, 0.1% NP40). PCR amplification and index incorporation were performed in a 50 μl reaction containing 5 μl of forward and reverse index primers (Illumina Nextera Index Kit), 15 μl NPM, 5 μl PPC (Illumina Nextera DNA Sample Preparation Kit) and 20 μl DNA. Libraries were purified using QIAquick PCR clean-up columns (QIAGEN) and sequenced on an Illumina HiSeq (6 libraries/lane).

QUANTIFICATION AND STATISTICAL ANALYSIS

RNA-seq reads were mapped to the mouse genome build NCBI37/mm9 using Bowtie2 and quantified using Seqmonk (Babraham Bioinformatics; https://www.bioinformatics.babraham.ac.uk/projects/seqmonk/). Differential expression analysis was performed using DESeq2 (Love et al., 2014), using all annotated genes, V_H genic transcripts and intergenic transcripts in a single analysis. Gene Set Enrichment Analysis (GSEA) was performed using GSEA 4.1 (Subramanian et al., 2005). Mouse gene names were

converted to human gene symbols, and ran with default parameters for genes with BaseMean > = 5. The Molecular Signature Database (MSigDB) hallmark gene sets and the transcription factor targets/regulatory target were used to perform pathway enrichment analysis limiting the output to the top 1000 gene sets.

For VDJ-seq, reads were mapped to the mouse genome build NCBI37/mm9 using Bowtie2. To quantify individual V and D genes, probes were created over each gene segment and correctly orientated reads were quantified over each probe using Seqmonk. Libraries were also analyzed using IMGT (International ImMunoGeneTics information system – http://www.imgt.org/ Lefranc et al., 2015). Analysis of VDJ-recombined sequences was carried out as described previously (Bolland et al., 2016); however, to analyze DJ-recombined sequences using IMGT, it was necessary to artificially add a V_H gene 5′ of the D_H sequence, as IMGT/HighVQUEST can only process VDJ sequences. The J558.78.182 V_H gene was appended, as it is functional and in-frame. These data were kept separate, and only D_H genes and the DJ_H junction were used for the analysis.

Due to the expectation for less variable VDJ events in IL-7Rα$^{-/-}$ cells, measures were taken to distinguish and discount technical duplicates in VDJ-seq libraries. VDJ-seq libraries were de-duplicated based on the sequence of read 2 (containing both V_H-DJ_H and D_H-J_H junctions) and the sequence and position of the V_H gene (LinkON pipeline described in Bolland et al. (2016). This method relies on the variability of gene usage, junction diversity, and the sonication step; therefore, reduced variability in the junctions and in V_H usage would lead to sequences being more likely to be identical, particularly in partially (D_H-J_H) recombined alleles. To overcome this issue, modified PE1 adaptors containing random barcodes were used to generate the IL-7Rα$^{-/-}$ and FL libraries, allowing PCR duplicates, biological duplicates and Illumina sequencing errors to be distinguished (Chovanec et al., 2018).

ATAC-seq reads were mapped to the mouse genome build NCBI37/mm9 using Bowtie2 and quantified using the MACS peak caller within Seqmonk. DESeq2 was used to identify genomic locations exhibiting significant differences in ATAC-seq reads in IL-7Rα/Rag2$^{-/-}$ relative to Rag2$^{-/-}$, and these sites were tested for TF motifs using the HOMER analysis tool (http://homer.ucsd.edu/homer/ngs/peaks.html - Heinz et al., 2010).

ChIP reads were mapped using Bowtie and peaks called using MACS2 (in the narrow peak mode): PAX5 Rag$^{-/-}$ pro-B (GSM932924) and EBF1 Rag$^{-/-}$ pro-B (GSM876622, GSM876623).

For calculating statistical significance between groups for the above datasets, a two-tailed ANOVA (type III) together with a pairwise t test (adjusted by Benjamini and Hochberg method) were used to calculate significance and p values (significant when > 0.05) when data were normal. When data failed the normality test a Kruskal-Wallis test (followed by pairwise Wilcox test (adjusted by Benjamini and Hochberg method) was performed. All results for specific tests are explained in the figure legends, including the statistical test used, value and meaning of n, and confidence intervals.

Cell Reports, Volume *36*

Supplemental information

IL-7R signaling activates widespread V_H and D_H gene usage to drive antibody diversity in bone marrow B cells

Amanda Baizan-Edge, Bryony A. Stubbs, Michael J.T. Stubbington, Daniel J. Bolland, Kristina Tabbada, Simon Andrews, and Anne E. Corcoran

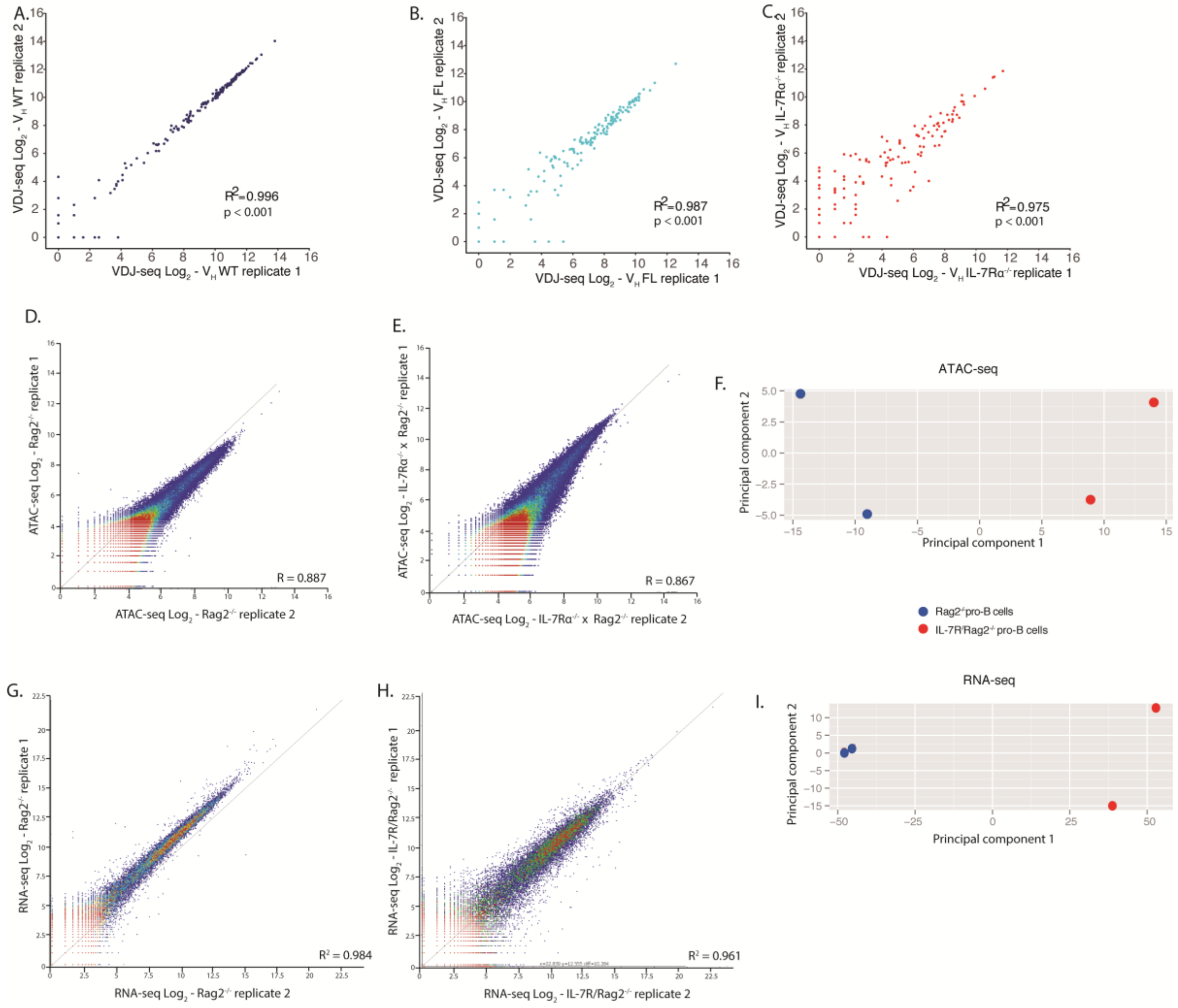

S1: Replicate VDJ-seq, ATAC-seq and RNA-seq libraries are highly correlated. Related to Figure 1 (panels A-C), 2 (panels A-C) and 3 (panels D-F and G-I).

A-C. Scatterplot of VDJ-seq read counts (log2 transformed) of V_H genes in two **A.** WT, **B.** FL and **C.** IL-7Rα$^{-/-}$ pro-B cell biological replicate datasets.

D-F. ATAC-seq read peaks were identified with MACS peak caller in Rag2$^{-/-}$ and IL-7Rα$^{-/-}$/Rag2$^{-/-}$ genotypes. Reads were quantified for peak probes for each biological replicate separately for **D.** Rag2$^{-/-}$ (Pearson's correlation R = 0.887) and **E.** IL-7Rα$^{-/-}$/Rag2$^{-/-}$ (R = 0.867) pro-B cell libraries. **F.** Principal component analysis shows clustering of each genotype. Principal components are calculated based on the regularized-logarithm transformation of read counts per gene and using the R package pcaMethods. Analysis done using DESeq2 package.

G-I. RNA-seq reads were quantified over all genes for each biological replicate for **G.** Rag2$^{-/-}$ and **H.** IL-7Rα$^{-/-}$/Rag2$^{-/-}$ pro-B cell libraries. **I.** Principal component analysis shows clustering of each genotype, calculated as for **F.** above.

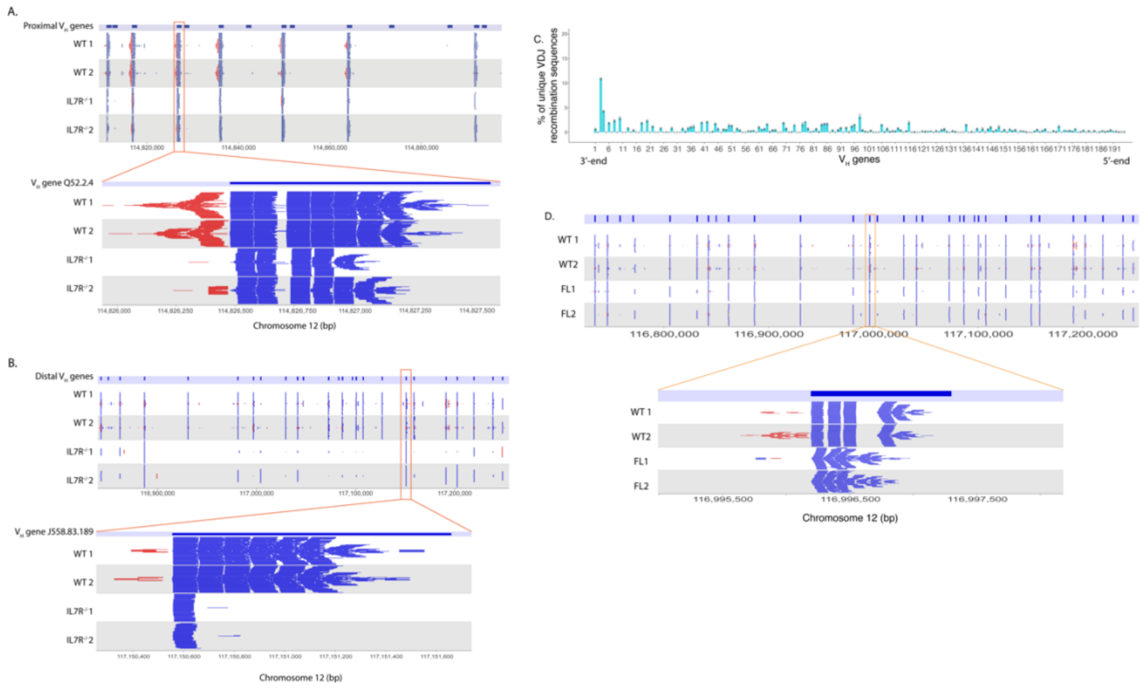

S2: VDJ-seq read depth view over the Igh V region. Related to Figure 1 and 2. Genome browser view of raw reads for each replicate WT and IL-7Rα$^{-/-}$ VDJ-seq library over the **A.** proximal V_H gene region (V_H genes in dark blue, starting with 7183.1pg.1 and 7183.8pg.14 as the most right) with a zoomed in view of Q52.2.4; and of the **B.** distal V_H gene region (the most left V_H genes represents J558.71pg.172 and the most right is J558.89pg.195) with a zoomed in view of J558.83.189. Due to the low number of cells in IL-7Rα$^{-/-}$ BM, VDJ-seq libraries were generated with approximately 6-fold less starting material than WT libraries, resulting in reduced numbers of raw reads (S Table 1). Nevertheless, this amount of starting material does not compromise detection of the wide dynamic range of frequency of VDJ and DJ recombined sequences (Chovanec et al., 2018). Blue reads = reverse, red reads = forward. Only correctly orientated (reverse) reads were quantified for VDJ-seq analysis.
C. Recombination frequencies of the 195 V_H genes measured by VDJ-seq, for FL pro-B cells from 15.5 day old wild-type mice (50 embryos per replicate). Two replicates are shown as open circles. Reverse-strand reads were quantified for each V_H gene and shown as a percentage of total number of reads quantified. For list of V_H genes see S Table 1. **D.** Genome browser view of raw reads for each replicate WT and FL VDJ-seq library over the distal V_H gene region (the most left V_H genes represents J558.66.165 and the most right is J558.89pg.195) with a zoomed in view of J558.75.177. Each WT and FL replicate is shown separately. For read depth mapped to each Igh region see S Table 1.

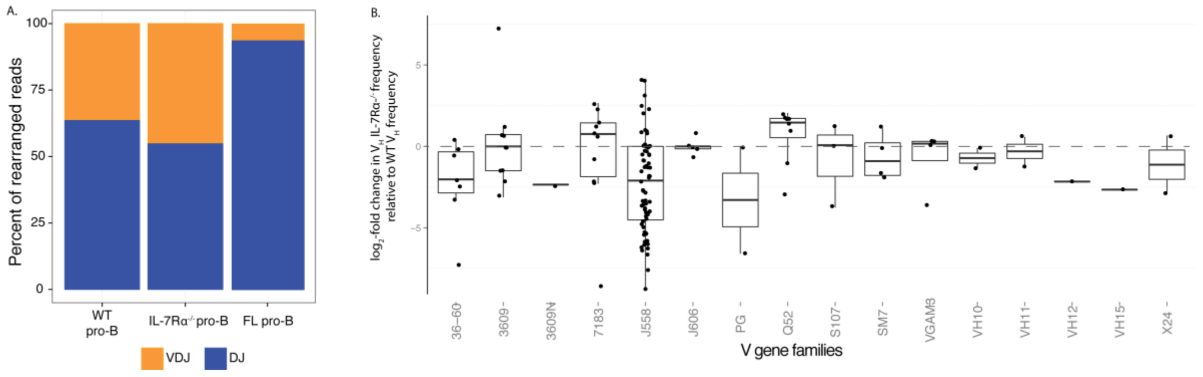

S3: DJ_H -VDJ_H ratio and individual V_H gene family usage. Related to Figure 1 and 2

A. Proportion of DJ_H and VDJ_H sequences in each cell type. The number of reads in the correct orientation was calculated over the D_H and V_H regions, and is shown as a percentage of total VDJ-seq reads in the correct orientation (mean of two replicates). **B.** VDJ-seq values of each V_H gene grouped in families. The average of two WT and two IL-7Rα$^{-/-}$ VDJ-seq replicates was calculated for each V_H gene. To display changes between WT and IL-7R-modified model frequencies, V_H frequencies for each model were divided with the WT mean value and log2-transformed for comparison between models. Log2 values for each gene were grouped by gene family.

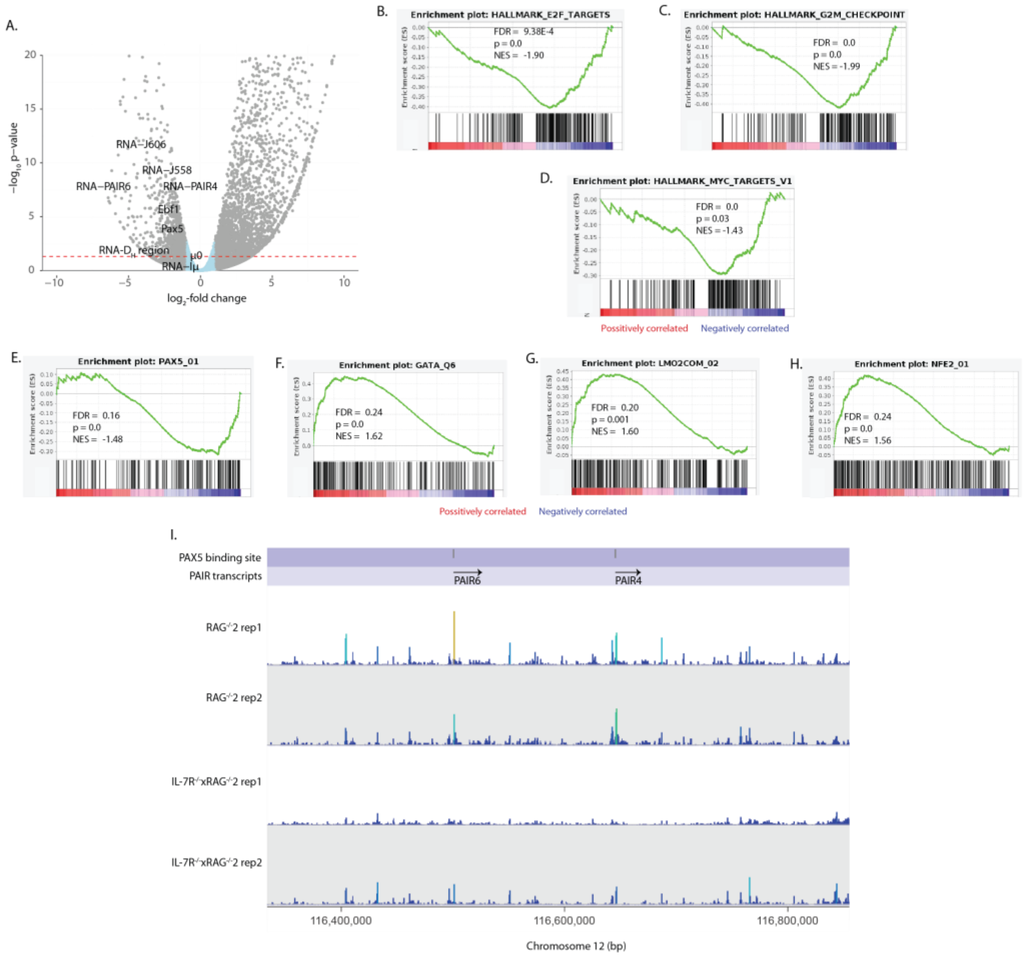

S4: Transcriptional changes in IL-7Rα/Rag2$^{-/-}$ pro-B cell libraries relative to Rag2$^{-/-}$. Related to Figure 4. **A.** Volcano plot showing genome-wide RNA-seq data. Grey dots are genes that are differentially expressed > 2-fold (in blue < 2-fold) in IL-7Rα$^{-/-}$/Rag2$^{-/-}$ compared with Rag2$^{-/-}$ libraries; dashed red line shows where p = 0.05. Transcripts of interest are tagged by name. Out of 4,793 differentially expressed genes in IL-7Rα/Rag2-/- pro-B compared with Rag2-/- pro-B cells, 3,780 genes were upregulated, while 1,013 genes were downregulated.

B-D. Gene set enrichment plot for **B.** E2F, **C.** G2M and **D.** MYC pathway target genes from the hallmark gene sets. Green line = enrichment score. FDR, false discovery rate; p, p-value; NES, normalized enrichment score.

E-H: Transcriptional profiles for lineage-specific TF in IL-7Rα$^{-/-}$/Rag2$^{-/-}$ pro-B cell. Gene set enrichment plot for **E.** PAX5, **F.** GATA, **G.** LMO2 and **H.** NFE2 pathway target genes from the transcription factor targets/regulatory target gene sets. Green line = enrichment score. FDR, false discovery rate; p, p-value; NES, normalized enrichment score.

I: PAIR4 and PAIR6 PAX5 binding sites have decreased accessibility in IL-7Rα$^{-/-}$Rag2$^{-/-}$ cells. ATAC-seq reads for Rag2$^{-/-}$ and IL-7Rα$^{-/-}$Rag2$^{-/-}$ were quantified per 500 bp bins along the Igh locus and normalised by reads per million in Seqmonk. Height and colour of each bar represents relative number of reads over each probe: high orange bars have more reads than short blue bars. Each track was generated from each ATAC-seq library replicate. Purple tracks represent the location of PAX5 binding sites (top) and the location of PAIR4 and PAI6 transcripts (black arrows, bottom).

Differentially expressed genes

Gene	Rag2$^{-/-}$ mean	IL-7Rα/Rag2$^{-/-}$ mean	log2 FoldChange	p-adj
Ebf1	198467.7	31632.21	-2.57	2.79E-07
Pax5	22013.66	5049.33	-2.05	5.39E-04
Flt3	371.14	2680.54	2.8	1.31E-14
Irf8	1727.72	7817.74	2.15	1.34E-09
Ikzf3	453.74	2896.93	2.58	6.89E-06
Lef1	50047.29	7152.81	-2.69	1.39E-05
Foxo1	19360.34	5176.08	-1.86	2.37E-05
Cd79a	36517.38	5427.5	-2.6	2.64E-04
Rag1	24672.56	4710.95	-2.26	2.37E-03
Smarca4	94899.66	25252.66	-1.84	3.19E-03
Ezh2	21004.9	8795.46	-1.23	3.77E-03
Myb	29838.38	16868.3	-0.8	2.41E-01
Irf4	5144.04	2515.98	-0.98	3.14E-01
Pdcd1	0	2.37	0.86	7.68E-01

Pax5 Igh

Gene	base Mean	log2Fold Change	stat	pvalue	padj	RAG2ko rep 1	RAG2ko rep 2	IL7RxRAG rep 1	IL7RxRAG rep 2	Start	End
PAX5_peak_4991	58.65	-1.607	-3.907	9.34E-05	0.003	104.98	71.196	21.862	36.603	116500485	116500825
PAX5_peak_4995	47.76	-1.757	-3.892	9.93E-05	0.003	65.817	81.8	13.991	29.461	116645504	116645995
PAX5_peak_4951	57.82	-1.249	-3.209	0.001	0.018	94.144	68.167	31.481	37.496	115420792	115421430
PAX5_peak_4980	80.22	-0.967	-2.937	0.003	0.034	109.14	103.01	50.719	58.029	116261760	116262315
PAX5_peak_4935	78.21	-1.018	-2.879	0.004	0.038	128.3	80.285	51.594	52.673	114559170	114559811
PAX5_peak_4962	55.66	-1.098	-2.689	0.007	0.056	94.977	56.048	32.355	39.281	115783152	115783717
PAX5_peak_4959	34.77	-1.226	-2.557	0.01	0.073	47.488	49.989	16.615	24.997	115731781	115732333
PAX5_peak_4994	20.89	-1.335	-2.214	0.026	0.135	32.492	27.266	13.991	9.82	116642334	116642774
PAX5_peak_5004	26.69	-1.164	-2.174	0.029	0.143	31.659	42.415	15.74	16.962	116893825	116894359
PAX5_peak_4997	23.16	-1.215	-2.074	0.038	0.169	41.656	22.722	13.991	14.284	116686368	116686763
PAX5_peak_4985	39.07	-0.908	-2.014	0.044	0.185	59.152	42.415	28.857	25.89	116403612	116404154
PAX5_peak_4946	27.56	-0.902	-1.696	0.089	0.289	44.156	27.266	20.987	17.855	115232421	115232908
PAX5_peak_4965	51.75	-0.66	-1.686	0.091	0.293	67.484	59.078	37.602	42.852	115848009	115848418
PAX5_peak_4961	33.38	-0.608	-1.255	0.209	0.472	42.49	37.87	32.355	20.533	115751308	115751839
PAX5_peak_4977	17.15	0.836	1.219	0.222	0.491	12.497	12.118	30.606	13.391	116229108	116229562
PAX5_peak_5010	31.94	0.614	1.219	0.222	0.491	27.493	22.722	48.096	29.461	117046284	117046584
PAX5_peak_4967	20.07	0.741	1.114	0.265	0.539	21.661	7.5741	34.104	16.962	115884627	115885044
PAX5_peak_4992	23.59	-0.546	-0.959	0.337	0.611	35.824	19.692	19.238	19.64	116550165	116550638
PAX5_peak_4973	18.22	-0.585	-0.935	0.349	0.623	22.494	21.207	12.242	16.962	116042685	116042985

PAX5_peak_4958	22.84	-0.53	-0.929	0.352	0.626	22.494	31.811	20.987	16.069	115707103	115707635
PAX5_peak_4953	15.15	-0.583	-0.826	0.408	0.671	12.497	24.237	9.619	14.284	115511658	115512198
PAX5_peak_5016	20.48	-0.497	-0.819	0.412	0.675	30.826	16.663	18.364	16.069	117238104	117238447
PAX5_peak_4976	43.69	-0.349	-0.796	0.425	0.687	36.658	62.107	36.728	39.281	116219764	116220117
PAX5_peak_4933	74.42	-0.249	-0.739	0.459	0.713	79.981	81.8	74.331	61.6	114466306	114466863
PAX5_peak_4998	19.18	0.423	0.689	0.49	0.736	17.495	15.148	25.359	18.748	116745638	116746002
PAX5_peak_5003	58.35	0.265	0.689	0.491	0.736	43.323	63.622	57.7158	68.742	116885865	116886170
PAX5_peak_4999	23.53	-0.388	-0.669	0.503	0.744	23.327	30.296	27.108	13.391	116757190	116757574
PAX5_peak_4966	47.54	0.258	0.628	0.53	0.765	39.99	46.959	58.59	44.638	115853579	115854300
PAX5_peak_4987	31.18	0.282	0.574	0.565	0.787	31.659	24.237	37.602	31.246	116431681	116432096
PAX5_peak_5014	15.78	0.343	0.514	0.606	0.813	14.163	13.633	18.364	16.962	117190501	117190981
PAX5_peak_5007	22.97	0.287	0.476	0.634	0.83	17.495	24.237	34.979	15.176	116929919	116930219
PAX5_peak_4934	34.32	0.205	0.402	0.687	0.86	41.656	21.207	27.108	47.316	114552752	114553205
PAX5_peak_5000	29.87	-0.11	-0.219	0.826	0.932	31.659	30.296	23.611	33.924	116765364	116765786
PAX5_peak_4990	21.76	0.1	0.176	0.859	0.946	20.828	21.207	23.611	21.426	116496004	116496536

ATAC-seq Igh

Gene	base Mean	log2Fold Change	stat	pvalue	padj	RAG2ko rep 1	RAG2ko rep 2	IL7RxRAG rep 1	IL7RxRAG rep 2
12_116500351..116501400	58.65	-1.607	-3.907	9.34E-05	0.002	104.975	71.196	21.862	36.603
12_116645251..116646300	47.76	-1.757	-3.892	9.93E-05	0.002	65.817	81.8	13.991	29.461
12_115420951..115421400	57.82	-1.249	-3.209	0.001	0.017	94.144	68.167	31.481	37.496
12_116261701..116262450	80.22	-0.967	-2.937	0.003	0.033	109.141	103.007	50.719	58.029
12_114559051..114559800	78.21	-1.018	-2.879	0.004	0.038	128.303	80.285	51.594	52.673
12_115783051..115783650	55.66	-1.098	-2.689	0.007	0.056	94.977	56.048	32.355	39.281
12_115731751..115732350	34.77	-1.226	-2.557	0.01	0.072	47.488	49.989	16.615	24.997
12_116893801..116894400	26.69	-1.164	-2.174	0.029	0.143	31.659	42.415	15.74	16.962
12_116403751..116404050	39.07	-0.908	-2.015	0.043	0.184	59.152	42.415	28.857	25.89
12_115847401..115848000	51.75	-0.661	-1.686	0.091	0.293	67.484	59.078	37.602	42.852
12_115751401..115751850	33.31	-0.609	-1.255	0.209	0.477	42.49	37.87	32.355	20.533
12_115825501..115825800	14.5	0.894	1.204	0.228	0.498	5.831	15.148	21.862	15.176
12_115850101..115850700	31.22	0.594	1.176	0.239	0.512	30.826	18.177	41.975	33.924
12_115884601..115885050	20.07	0.741	1.114	0.265	0.538	21.661	7.574	34.104	16.962
12_116550151..116550450	23.59	-0.546	-0.959	0.337	0.611	35.824	19.692	19.238	19.64
12_115707001..115707600	22.84	-0.53	-0.929	0.352	0.626	22.494	31.811	20.987	16.069
12_116219551..116220450	43.69	-0.349	-0.796	0.425	0.689	36.658	62.107	36.728	39.281
12_115617001..115617600	38.55	-0.33	-0.705	0.48	0.728	49.155	36.355	43.724	24.997
12_116885701..116886450	58.35	0.265	0.689	0.49	0.735	43.323	63.622	57.715	68.742
12_116757301..116757600	23.53	-0.389	-0.669	0.503	0.744	23.327	30.296	27.108	13.391
12_115853401..115854450	47.54	0.258	0.628	0.53	0.764	39.99	46.959	58.59	44.638
12_116431651..116432250	31.18	0.282	0.5746	0.565	0.786	31.659	24.237	37.602	31.246
12_116929951..116930250	22.97	0.287	0.476	0.634	0.83	17.495	24.237	34.979	15.176
12_116765401..116765850	29.87	-0.11	-0.219	0.826	0.931	31.659	30.296	23.611	33.924

S Table 3: EBF1, PAX5 and their key targets are differentially expressed and accessible in IL-7R$\alpha^{-/-}$ Rag2$^{-/-}$ relative to Rag2$^{-/-}$ pro-B cells. Related to Figure 4 and Figure S4. **Differentially expressed genes.** RNA-seq raw reads were quantified over all genes, separately for each of two replicates. The adjusted mean for each probe, as well as the log2-fold change and adjusted p-value in the IL-7R$\alpha^{-/-}$ Rag2$^{-/-}$ relative to Rag2$^{-/-}$ samples was calculated by DESeq2. **Pax5 Igh. Pax5 binding sites show changes in accessibility in IL-7R$\alpha^{-/-}$ Rag2$^{-/-}$ relative to Rag2$^{-/-}$ pro-B cells.** ATAC-seq raw reads were quantified over PAX5 binding sites (only PAX5 binding sites over the Igh locus are shown) for each of two replicates. The adjusted mean for each probe, as well as the log2-fold change and adjusted p-value in the IL-7R$\alpha^{-/-}$ Rag2$^{-/-}$ relative to Rag2$^{-/-}$ samples was calculated by DESeq2. **ATAC-seq Igh.** Location of 24 accessible sites in Igh.
